# Australian Assassins, Part III: A review of the Assassin Spiders (Araneae, Archaeidae) of tropical north-eastern Queensland

**DOI:** 10.3897/zookeys.218.3662

**Published:** 2012-08-30

**Authors:** Michael G. Rix, Mark S. Harvey

**Affiliations:** 1 Department of Terrestrial Zoology, Western Australian Museum, Locked Bag 49, Welshpool DC, Perth, Western Australia 6986, Australia; 2 Research Associate, Division of Invertebrate Zoology, American Museum of Natural History, New York, NY 10024, USA; 3 Research Associate, California Academy of Sciences, 55 Music Concourse Drive, San Francisco, CA 94118, USA; 4 Adjunct Professor, School of Animal Biology, University of Western Australia, 35 Stirling Highway, Crawley, Perth, Western Australia 6009, Australia

**Keywords:** New species, taxonomy, conservation, Wet Tropics, rainforests, Palpimanoidea

## Abstract

The assassin spiders of the family Archaeidae from tropical north-eastern Queensland are revised, with eight new species described from rainforest habitats of the Wet Tropics bioregion and Mackay-Whitsundays Hinterland: *Austrarchaea griswoldi* **sp. n.**, *Austrarchaea hoskini* **sp. n.**, *Austrarchaea karenae* **sp. n.**, *Austrarchaea tealei* **sp. n.**, *Austrarchaea thompsoni* **sp. n.**, *Austrarchaea wallacei* **sp. n.**, *Austrarchaea westi* **sp. n.** and *Austrarchaea woodae* **sp. n.** Specimens of the only previously described species, *Austrarchaea daviesae* Forster & Platnick, 1984, are redescribed from the southern Atherton Tableland. The rainforests of tropical eastern Queensland are found to be a potential hotspot of archaeid diversity and endemism, with the region likely to be home to numerous additional short-range endemic taxa. A key to species complements the taxonomy, with maps, natural history information and conservation assessments provided for all species.

## Introduction

Few families of Australian spiders are as distinctive or as enigmatic as the ‘assassin spiders’ of the family Archaeidae, renowned for their unique cephalic morphology, strange araneophagic biology, great phylogenetic antiquity and relictual biogeography across the Southern Hemisphere. Although once considered to be one of the rarest of spiders families – and certainly one of the least understood in terms of taxonomy, phylogeny and biology – recent and ongoing research in the U.S.A., South Africa and Australia has shed increasing light on this remarkable lineage of spiders (see Platnick 1991a, 1991b, Lotz 1996, 2003, 2006, Harvey 2002a, Griswold et al. 2005, Wood et al. 2007, Wood 2008, Rix and Harvey 2011, 2012a, 2012b, Wood et al. in press). Indeed, archaeids are now one of the better understood families of Araneae, with the southern African and Malagasy faunas continuing to be revised and phylogenetically tested, and the Australian fauna now almost completely revised taxonomically and phylogenetically. The last 20 years have seen large numbers of new species discovered and described in both the Afro-Malagasy and Australian regions (Platnick 2012), and archaeids (and their relatives) continue to feature heavily in studies exploring the classification and phylogeny of the basal Araneomorphae (e.g. Schütt 2000, Griswold et al. 2005, Rix et al. 2008, Wood et al. in press).

Australian Archaeidae have been progressively revised over the last two years, with 26 new species described since 2011, taking the total number of currently described Australian species to 30 (Platnick 2012). Rix and Harvey (2011) first documented the Archaeidae of ‘mid-eastern’ Australia, re-describing the only previously named species from the region, and describing 17 new species from south-eastern Queensland and eastern New South Wales. This revision first noted the possibility of two major taxonomic, phylogenetic and biogeographic disjunctions in eastern and southern Australia, highlighting the St Lawrence Gap (Webb and Tracey 1981) in central eastern Queensland, and the Australian Alps in south-eastern Australia as likely candidates (see Fig. 2). Subsequent molecular phylogenetic research by Rix and Harvey (2012b) tested and further confirmed these phylogenetic disjunctions, highlighting especially the allopatric separation of a deeply-divergent southern Australian (i.e. Victorian, South Australian and Western Australian) lineage from all other eastern Australian taxa. Rix and Harvey (2012b) also confirmed the importance of the St Lawrence Gap, between Gladstone and Mackay, as an important phylogenetic and biogeographic barrier between monophyletic clades of *Austrarchaea* Forster & Platnick, 1984 from tropical north-eastern Queensland and mid-eastern Australia (see Figs 2–3); a result congruent with morphological observations by Rix and Harvey (2011, figs 5E–G). A second taxonomic revision by Rix and Harvey (2012a) formally documented the distinctive southern Australian lineage, describing the new genus *Zephyrarchaea* Rix & Harvey, 2012a, along with nine new species from south-western Western Australia, Kangaroo Island (South Australia) and southern Victoria. As a result of these monographic and phylogenetic revisions, a surprisingly diverse Australian archaeid fauna has now been revealed, one dominated by large numbers of mostly allopatric, short-range endemic species, all of which are restricted to the ‘mesic zone’ (Byrne et al. 2011) of mainland Australia. Archaeids are ubiquitous in the tropical and subtropical upland rainforests of eastern Australia, and only those species from north of the St Lawrence Gap remain to be revised (Fig. 2).

The current paper – the third and final in a series revising the Archaeidae of Australia – presents a taxonomic revision of the assassin spiders from tropical north-eastern Queensland, including those species from the Mackay-Whitsundays Hinterland and the Wet Tropics bioregion, between Cooktown and Townsville (Figs 2, 25). This revision takes the total number of described Australian Archaeidae to 38 species, with the genus *Austrarchaea* now including 27 described short-range endemic species.

## Material and methods

All taxa were described and illustrated from specimens stored in 75% or 95% ethanol. Digital images were taken using a Leica MZ16A binocular microscope and a Leica DM2500 compound microscope, with auto-montage images captured using Leica DFC500 mounted cameras with Leica Application Suite Version 3.6.0 software. Male left pedipalps were dissected prior to imaging and bulbs were aligned for standardised comparison in the ventral and retrolateral positions illustrated. Female genitalia were dissected and cleared in a 10% lactic acid plus 90% glycerol solution, prior to mounting on temporary glass slides and imaging in a postero-ventral position (Fig. 14G; see also Rix and Harvey 2011, fig. 5D), usually with genital plates removed (Fig. 7G). This postero-ventral alignment allowed for a much clearer presentation of the spermathecae, while still permitting inter-specific comparison at an equivalent (albeit opposite) plane to that of Rix and Harvey (2011, 2012a). Illustrations were made on Utoplex tracing paper, using printed template auto-montage images. Maps were generated using ArcMap version 9.3.1 (ESRI Inc.) with Virtual Earth (Microsoft Corp.).

Measurements are in millimetres (rounded to the nearest hundredth of a millimetre) and were taken using an ocular graticule on a Leica M80 binocular microscope. Left legs were removed from specimens prior to taking measurements and imaging lateral body profiles. Lateral profile images were standardised for inter-specific comparison by vertically aligning the centre of each left anterior median eye with the lower anterior margin of the carapace (above the labrum) (Rix and Harvey 2011, 2012a). Carapace height was measured in lateral view, from the margin of the pars thoracica above coxa II to the highest point of the pars cephalica (Fig. 5). Carapace length was measured from the lower anterior margin of the carapace (above the labrum) to the posterior margin of the pars thoracica (above the pedicel) (Fig. 5). ‘Neck’ width was measured in lateral view, at the narrowest point of the carapace, with total length, carapace width, abdomen length and abdomen width all measured in dorsal view. To quantify inter-specific variation in the shape of the cephalothorax and ‘head’, three morphometric ratios (the *carapace height to carapace length* [CH/CL] *ratio*; the *post-ocular ratio* [P.O. ratio]; and the *highest point of pars cephalica* [HPC] *to post-ocular length ratio*) were derived from lateral profile images as defined and discussed by Rix and Harvey (2011, 2012a). For Material Examined sections, specimens not examined for the current revision, but currently housed at (or on loan to) the California Academy of Sciences (due to ongoing research) are listed separately, with identifications confirmed by H. Wood; one additional, unseen vial (QMB S50322) was identified as *Austrarchaea daviesae* according to geographic proximity (Fig. 16). Species descriptions and numbering of the pedipalpal sclerites follows Rix and Harvey (2011) (but see also Remarks, below).

**Abbreviations used in the text are as follows:**

CH/CL Carapace height (CH) to carapace length (CL) ratio

F1/CL Femur I length (F1) to carapace length (CL) ratio

HPC Highest point of pars cephalica

HT 1-4 Abdominal hump-like tubercles 1-4

SEM Scanning electron micrograph/s

TS 1-3 Tegular sclerites 1-3

**Specimens described in this study are lodged at the following institutions:**

AMS Australian Museum, Sydney (G. Milledge)

ANIC Australian National Insect Collection, Canberra (B. Halliday)

CASENT California Academy of Sciences, San Francisco (C. Griswold, A. Carmichael)

QMB Queensland Museum, Brisbane (R. Raven, O. Seeman)

WAM Western Australian Museum, Perth (MSH, J. Waldock)

## Taxonomy

### Family Archaeidae Koch & Berendt, 1854

#### Genus Austrarchaea

Forster & Platnick, 1984

http://species-id.net/wiki/Austrarchaea

Austrarchaea Forster & Platnick, 1984: 21; Rix and Harvey 2011: 14.

##### Type species.

*Archaea nodosa* Forster, 1956, by original designation.

**Diagnosis.** Species of *Austrarchaea* can be distinguished from all southern Australian species of *Zephyrarchaea* by the significantly taller carapace (CH/CL ratio ≥ 2.0), by the presence of accessory setae on the distal bulge of the male cheliceral paturon, and by the fusion of the two conductor sclerites on the male pedipalp (Rix and Harvey 2012a, fig. 4). Australian Archaeidae are further distinguished from Old World taxa by the presence of numerous, clustered spermathecae in females (Fig. 7G), and by the presence of a long, wiry embolus on the pedipalp of males (Fig. 4).

##### Description.

For a full generic description see Rix and Harvey (2011). For notes on genitalia and morphological differences among lineages of *Austrarchaea*, see Remarks (below).

##### Distribution.

**Figure 1.**
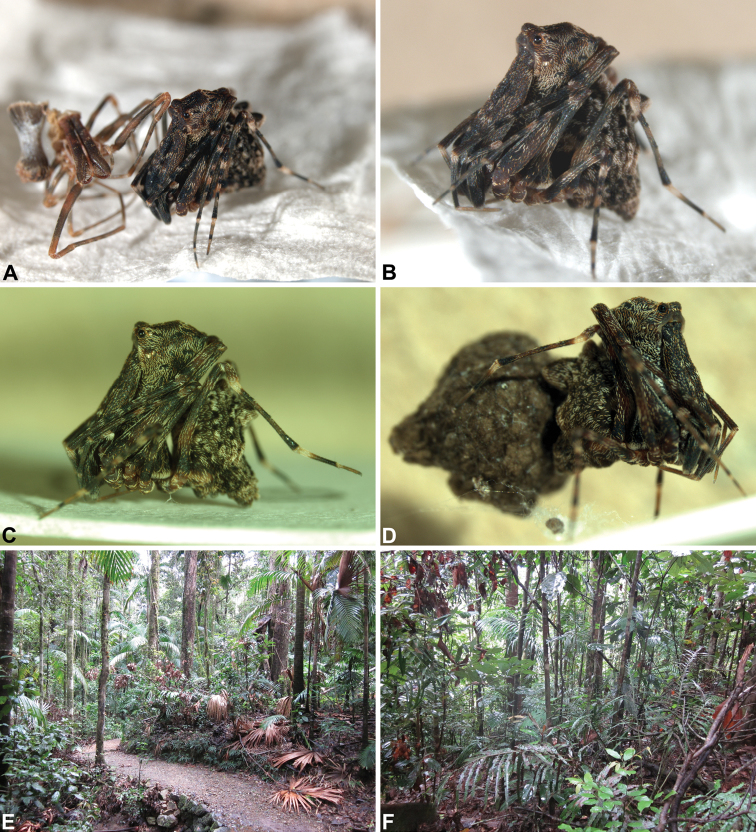
Habitus and habitat images of species Archaeidae from north-eastern Queensland. **A–D**, Habitus images of live paratype specimens of *Austrarchaea griswoldi* sp. n. from Eungella National Park: **A** newly-moulted female with recently cast cuticle; **B–C**, female, lateral view; **D**, female carrying egg-sac. **E–F**, Habitat images: **E**, tropical rainforest at Broken River, Eungella National Park – type locality of *Austrarchaea griswoldi* sp. n.; **F**, dense tropical rainforest at Malaan National Park, Atherton Tableland – locality of *Austrarchaea daviesae* Forster & Platnick.

**Figure 2.**
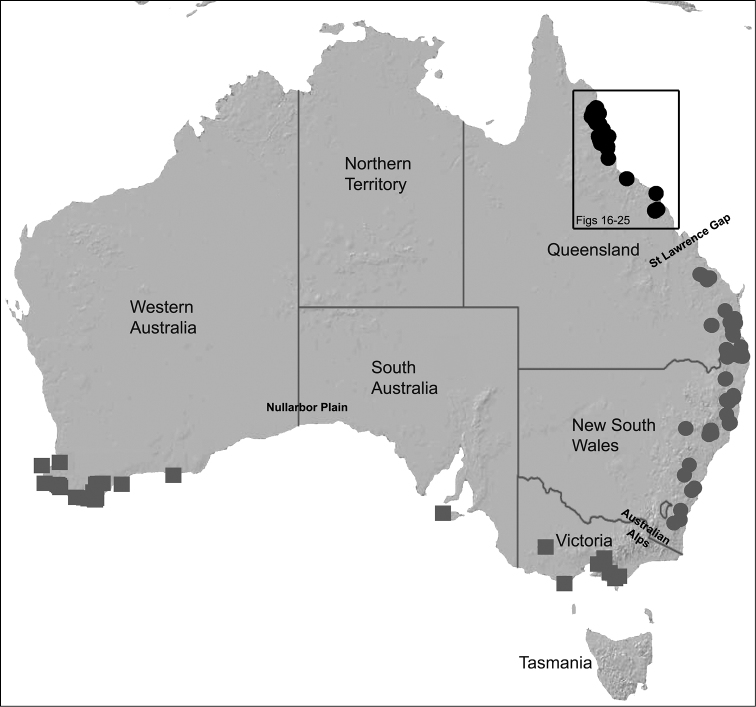
Map showing the known distribution of Archaeidae in Australia (circles for the genus *Austrarchaea*; squares for *Zephyrarchaea*), with locality records for north-eastern Queensland species of *Austrarchaea* in the *Austrarchaea daviesae* species-group highlighted in black. Note the three major biogeographic and phylogenetic disjunctions in the distribution of Australian Archaeidae (see Rix and Harvey 2012b), especially the St Lawrence Gap, separating mid-eastern Australian taxa in the *Austrarchaea nodosa* species-group (see Figs 3–4).

**Figure 3.**
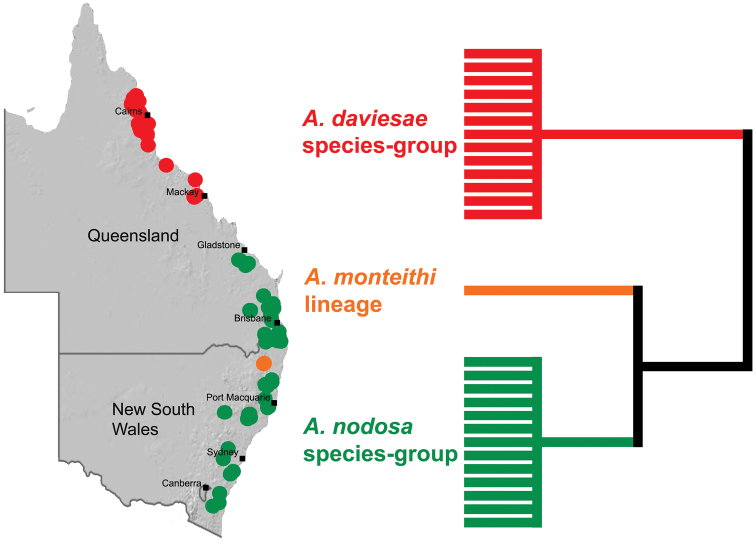
Distribution and phylogeny of *Austrarchaea* species from Rix and Harvey (2011, 2012b), showing the interrelationships of the three lineages from north-eastern Queensland (*Austrarchaea daviesae* species-group), mid-eastern Australia (*Austrarchaea nodosa* species-group) and the Gibraltar Range (*Austrarchaea monteithi* lineage), respectively. See Figure 4 for a comparison of morphological differences between these three clades.

Species of *Austrarchaea* occur in mesic habitats throughout eastern Queensland and New South Wales (Fig. 3), usually in montane rainforests (Figs 1E-F), but also in lowland rainforests or wet eucalypt forests on or adjacent to the Great Dividing Range (Rix and Harvey 2011). In north-eastern Queensland, archaeids occur throughout the Wet Tropics bioregion, from the Mount Finnigan Uplands (near Cooktown) south to Mount Elliot (near Townsville) (Figs 16–23, 25). In the Mackay and Whitsundays Hinterland region, archaeids can be found in the Eungella National Park (near Mackay), north to Mount Dryander (south of Bowen) (Figs 24–25). The genus is not known to occur south or west of the Australian Alps (Fig. 2), which may be a vicariant biogeographic barrier between populations of *Austrarchaea* and *Zephyrarchaea* (Rix and Harvey 2012a, 2012b).

##### Composition.

Nineteen described species – *Austrarchaea alani* Rix & Harvey, 2011, *Austrarchaea aleenae* Rix & Harvey, 2011, *Austrarchaea binfordae* Rix & Harvey, 2011, *Austrarchaea christopheri* Rix & Harvey, 2011, *Austrarchaea clyneae* Rix & Harvey, 2011, *Austrarchaea cunninghami* Rix & Harvey, 2011, *Austrarchaea daviesae* Forster & Platnick, 1984, *Austrarchaea dianneae* Rix & Harvey, 2011, *Austrarchaea harmsi* Rix & Harvey, 2011, *Austrarchaea helenae* Rix & Harvey, 2011, *Austrarchaea judyae* Rix & Harvey, 2011, *Austrarchaea mascordi* Rix & Harvey, 2011, *Austrarchaea mcguiganae* Rix & Harvey, 2011, *Austrarchaea milledgei* Rix & Harvey, 2011, *Austrarchaea monteithi* Rix & Harvey, 2011, *Austrarchaea nodosa* (Forster, 1956), *Austrarchaea platnickorum* Rix & Harvey, 2011, *Austrarchaea raveni* Rix & Harvey, 2011, *Austrarchaea smithae* Rix & Harvey, 2011 – plus the eight new species from north-eastern Queensland: *Austrarchaea griswoldi* sp. n., *Austrarchaea hoskini* sp. n., *Austrarchaea karenae* sp. n., *Austrarchaea tealei* sp. n., *Austrarchaea thompsoni* sp. n., *Austrarchaea wallacei* sp. n., *Austrarchaea westi* sp. n. and *Austrarchaea woodae* sp. n.

##### Remarks.

**Figure 4.**
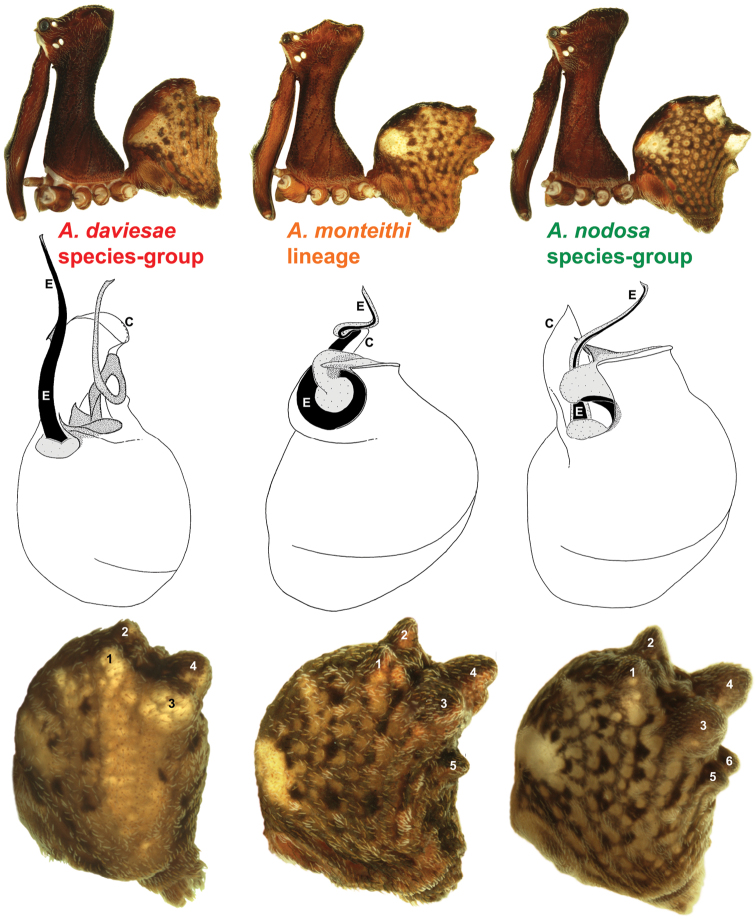
Morphological differences between the three lineages of *Austrarchaea* (see Fig. 3). Note the variation in the shape of the male pedipalp and the marked differences in the shape and orientation of the conductor (C), embolus (E) and the distal tegular sclerites. Note also the number of abdominal hump-like tubercles (1-6): four in the *Austrarchaea daviesae* species-group; five in *Austrarchaea monteithi*; and six in the *Austrarchaea nodosa* species-group.

The genus *Austrarchaea* includes three major lineages in eastern Australia (Figs 3–4), each readily distinguished by the morphology of the abdomen and the structure of the male pedipalp (Fig. 4). The most widespread lineage (the *Austrarchaea nodosa* species-group) occurs south of the St Lawrence Gap, from Kroombit Tops National Park in central Queensland, south to the Badja State Forest in southern New South Wales (Fig. 3); species in this lineage possess six dorsal hump-like tubercles on the abdomen and an exposed tegular cavity with a variably scutiform conductor (Fig. 4). The second, most restricted lineage (the *Austrarchaea monteithi* lineage) is known only from the Gibraltar Range National Park in northern New South Wales (Fig. 3); the single known species, *Austrarchaea monteithi*, possesses five dorsal hump-like tubercles on the abdomen and an exposed tegular cavity with a hooked conductor (Fig. 4). The third lineage (the *Austrarchaea daviesae* species-group; revised in this paper) occurs north of the St Lawrence Gap, from Eungella National Park north to Cooktown (Figs 3, 25); species in this lineage possess only four dorsal hump-like tubercles on the abdomen (recumbent in *Austrarchaea woodae* sp. n.) and a more enclosed tegular cavity with a very large, arched conductor (Figs 4, 6–15).

Although the derived pedipalpal morphology of *Austrarchaea daviesae* and its relatives is strikingly different to that of congeners further south, the distal tegular sclerites can nonetheless be broadly homologised with those of *Austrarchaea nodosa* and *Austrarchaea monteithi* on the basis of their shape and relative position in the unexpanded tegular cavity. The embolus in all nine known north-eastern Queensland species is a long, sinuous, strongly sclerotized process emerging from the distal bulb pro-ventrally, in some species bearing an additional accessory spur. Tegular sclerite 3 (TS 3) is always a prominent, pro-ventrally directed process, which is fused to the retro-ventral margin of the tegular bulb (the latter of which is usually also concomitantly modified). Tegular sclerite 2 (plus 2a, i.e. TS 2-2a) is usually inserted just behind TS 3 in the unexpanded tegular cavity, forming a distinctive, mesally-looped and distally whip-like structure common to all taxa in the *Austrarchaea daviesae* species-group; the extent of this very long, whip-like TS 2a is usually proximate to the distal extension of the embolus in the unexpanded state. This TS 2-2a morphology is in stark contrast to that of *Austrarchaea monteithi*, *Austrarchaea nodosa* and related species, in which TS 2a is usually covered and largely obscured by a more spur-like TS 2 process. Tegular sclerite 1 (TS 1) – generally the most prominent sclerite in species of *Zephyrarchaea* and other species of *Austrarchaea* – is reduced and often obscured in most archaeid species from north-eastern Queensland, although a few taxa possess a larger, more distinctive TS 1 posterior to the TS 2-2a complex (e.g. Fig. 9D). Inter-specific variation among taxa in the *Austrarchaea daviesae* species-group is pronounced, with male pedipalp morphologies usually highly autapomorphic for each species. Five broad pedipalp types (Types A-E) can be distinguished among north-eastern Queensland taxa, with Type A being the most common form, shared between five of the nine known species, and Types B-E each currently unique to single species. Figure 6 highlights differences between these different pedipalp morphologies, which are further diagnosed in the Key to species (see below).

##### Key to the species of *Austrarchaea* known from north-eastern Queensland (males required)

**Table:** 

1	Distal embolus enclosed within conductor (Fig. 12D); pedipalp very small, width of bulb << 0.30 mm (Fig. 12D) (**Type B** pedipalp; Fig. 6)	*Austrarchaea westi* sp. n.
–	Distal embolus fully exposed, projecting distally, not enclosed within conductor (Figs 7E, 13D, 15E); pedipalp larger, width of bulb > 0.30 mm	2
2	Conductor arched, directed prolaterally in ventral view (Fig. 14E); tegular sclerite 3 (TS 3) very large, dagger-like, directed pro-ventrally across bulb (Figs 14E-F); embolus with prominent, rounded, fin-shaped spur (Fig. 14E) (**Type D** pedipalp; Fig. 6)	*Austrarchaea hoskini* sp. n.
–	Conductor directed retrolaterally in ventral view (Figs 7E, 8D, 10D); tegular sclerite 3 (TS 3) not dagger-like; embolic spur, if present, with pointed apex (Figs 9E, 11F)	3
3	Distal bulb and proximal conductor strongly constricted laterally, forming uniquely apple-shaped pedipalpal profile in ventral view (Figs 13C–D); tegular sclerite 3 (TS 3) large, flattened, with prominent, distally folded apex (Figs 13D-E) (**Type C** pedipalp; Fig. 6)	*Austrarchaea woodae* sp. n.
–	Distal bulb and proximal conductor not constricted laterally; tegular sclerite 3 (TS 3) not folded distally	4
4	Ventro-distal rim of tegulum distally extended to form rectangular opercular plate, covering tegular sclerite 2a (TS 2a) for most of its length (Fig. 15E); tegular sclerite 3 (TS 3) very large, flattened, extending along entire retrolateral edge of conductor (Fig. 15F) (**Type E** pedipalp; Fig. 6)	*Austrarchaea griswoldi* sp. n.
–	Ventro-distal rim of tegulum not forming rectangular opercular plate; tegular sclerite 3 (TS 3) shorter, spur-like (Figs 7E, 8D, 9D, 10D, 11E); conductor arched, directed retrolaterally in ventral view, not abutting TS 3 (Figs 7E, 9D, 10D, 11E) (**Type A** pedipalp; Fig. 6)	5
5	Embolus with triangular embolic spur (Figs 8D, 9E, 10D, 11F); embolus projecting beyond distal rim of conductor by > 1/3 length of exposed embolic portion (Figs 9D, 10D, 11E)	6
–	Embolus without embolic spur (Fig. 7E); embolus projecting beyond distal rim of conductor by ~1/3 length of exposed embolic portion (Figs 7D-E)	*Austrarchaea daviesae* Forster & Platnick, 1984
6	Embolic spur distally positioned, situated close to pro-distal margin of conductor (slightly proximal to distal-most curve of embolus tip) (Figs 9D, 11E); tegular sclerite 1 (TS 1) relatively large, triangular, visible in ventral view posterior to TS 2-3 (Figs 8D, 9D, 11E)	7
–	Embolic spur more proximally positioned, situated near base of exposed embolic portion (Fig. 10D); tegular sclerite 1 (TS 1) small, obscured by TS 2-3, not visible in ventral view (Fig. 10D)	*Austrarchaea thompsoni* sp. n.
7	Tegular sclerite 3 (TS 3) with sharply pointed, claw-like apex (Figs 9D–E, 11E-F)	8
–	Tegular sclerite 3 (TS 3) with more bluntly pointed, triangular apex (Figs 8C–D)	*Austrarchaea wallacei* sp. n.
8.	Tegular sclerite 1 (TS 1) broadly triangular in ventral view (Fig. 9D); tegular sclerite 3 (TS 3) with single, sharply pointed process distally (Fig. 9D)	*Austrarchaea karenae* sp. n.
–	Tegular sclerite 1 (TS 1) with more tapered, tooth-like triangular apex in ventral view (Fig. 11E); tegular sclerite 3 (TS 3) with second short, pointed process distally (Fig. 11E)	*Austrarchaea tealei* sp. n.

### The Wet Tropics fauna

#### Austrarchaea daviesae

Forster & Platnick, 1984

http://species-id.net/wiki/Austrarchaea_daviesae

Figs 7 16 25

Austrarchaea daviesae Forster & Platnick, 1984: 22, figs 66–68, 70–75.

##### Vernacular name.

Misty Mountains Assassin Spider

##### Type material.

Holotype male: Majors Mountain, [Tully Falls National Park], Atherton Tableland, Queensland, Australia, [17°38'25"S, 145°32'14"E], collected at night, 14–20.IV.1978, V. Davies, R. Raven (QMB S1091).

Paratypes: Allotype female, “Malaan State Forest” [= Malaan National Park], Atherton Tableland, Queensland, Australia, [17°35'S, 145°35'E], 20–24.IV.1978, V. Davies, R. Raven (QMB S1092).

##### Other material examined.

**AUSTRALIA: *Queensland*: Tully Falls National Park (Atherton Tableland):** Massey Creek, 17°37'S, 145°34'E, flight intercept trap, 1000 m, 2–30.V.1996, P. Zborowski, 1♀ (ANIC). **Malaan National Park (Atherton Tableland):** “Malaan State Forest”, on Highway, 17°35'S, 145°35'E, pitfall trap, 850 m, 7.III.–15.V.1995, G. Monteith, J. Hasenpusch, 1 juvenile (QMB S38624); Mount Fisher, 7 km SW. of Millaa Millaa, pyrethrum knockdown, 1050–1100 m, 27–29.IV.1982, G. Monteith, D. Yeates, D. Cook, 1 juvenile (QMB S30838); next to Old Palmerston Highway, opposite Biggs Road, SSW. of Millaa Millaa, 17°35'11"S, 145°34'57"E, sifting elevated leaf litter at base of lawyer vine palms, tropical rainforest, 969 m, 18.III.2012, M. & A. Rix, 1♂, 1♀ (WAM T125183). **Wooroonooran National Park:** Mount Bartle Frere, inside Upper Boulder Caves, 17°23'S, 145°47'E, 1000 m, 12.V.1995, G. Monteith, D. Slaney, 1♀ (QMB S72989); same data except outside Lower Boulder Caves, 900 m, 13.V.1995, 1♀ (QMB S72987).

##### Other material (not examined).

**AUSTRALIA: *Queensland*: Atherton Tableland:** Bally Knob, summit, 17°39'S, 145°30'E, flight intercept trap, 1100 m, 6.XII.1998–6.II.1999, G. Monteith, D. Cook, 2♀ (QMB S50332). **Wooroonooran National Park:** Mount Bartle Frere, on track to summit, western side, from Junction Camp carpark off Gourka Road, 17°22'42"S, 145°47'09"E, day collecting, beating high and low vegetation, rainforest, 700–1300 m, 23–26.IV.2009, H. Wood, 3♂, 1♀ (CASENT 9034523); same data, 1♂ (CASENT 9034522); same data, 1 juvenile (CASENT 9034511); same data except day collecting, sifting leaf litter and small logs, brushing logs, mini-winkler, 1♀ (CASENT 9028381); Mount Bartle Frere, 18.4 km E. of Malanda, 17°22'46"S, 145°45'46"E, rainforest, 690–800 m, 17.III.2006, C. Griswold, D. Silva, M. Ramírez, 1 juvenile (CASENT 9023672).

##### Diagnosis.

*Austrarchaea daviesae* can be distinguished from all other Archaeidae from north-eastern Queensland by the absence of a spur on the embolus (Fig. 7E) combined with a Type A pedipalp morphology (Fig. 6), i.e. with a large, arched, retrolaterally directed conductor (Figs 6, 7E), exposed embolus (Figs 6, 7E) and relatively short, spur-like tegular sclerite 3 (TS 3). This species can be further distinguished by the unique shape of TS 3, which has a broad tegular base and strongly hooked apex (Figs 7D-F; see also Forster and Platnick 1984, figs 70–72, 74), and by the relatively short embolus, which projects beyond the distal rim of the conductor by ~1/3 the length of the exposed embolic portion (Figs 7D–E).

##### Description.

*Holotype male*: Total length 2.74; leg I femur 2.73; F1/CL ratio 2.38. Cephalothorax tan-brown; legs pale tan-brown with darker annulations; abdomen mottled tan-brown and yellowish-beige (colour faded due to preservation) (Fig. 7B). Carapace tall (CH/CL ratio 2.08); 1.15 long, 2.38 high, 1.08 wide, ‘neck’ 0.62 wide; bearing two pairs of rudimentary horns; highest point of pars cephalica (HPC) near posterior third of ‘head’ (ratio of HPC to post-ocular length 0.67), carapace gently sloping posterior to HPC; ‘head’ not strongly elevated dorsally (post-ocular ratio 0.27). Chelicerae with short brush of accessory setae on anterior face of paturon (Fig. 7C). Abdomen 1.54 long, 1.03 wide; with two pairs of dorsal hump-like tubercles (HT 1-4); dorsal scute fused anteriorly to epigastric sclerites, extending posteriorly to first pair of hump-like tubercles; HT 3-4 each covered by separate dorsal sclerites. Unexpanded pedipalp (of WAM T125183) (Figs 7D–F; see Forster and Platnick 1984, figs 70–74 for SEM images of unexpanded holotype pedipalp) of Type A morphology (Fig. 6), with large, retrolaterally directed, arched conductor; embolus distally directed, slightly sinuous, without spur, projecting beyond distal rim of conductor by ~1/3 length of exposed embolic portion; tegular sclerite 3 (TS 3) short, spur-like, with broad tegular base and strongly hooked apex; TS 2-2a looped over retrolateral edge of conductor, TS 2 not strongly developed distally, TS 2a projecting beyond distal rim of conductor to just past tip of embolus; TS 1 very small, obscured by TS 2-3, not visible in ventral view.

*Female* (WAM T125183): Total length 3.44; leg I femur 2.97; F1/CL ratio 2.32. Cephalothorax dark reddish-brown; legs tan-brown with darker annulations; abdomen mottled dark grey-brown and beige (Fig. 7A). Carapace tall (CH/CL ratio 2.11); 1.28 long, 2.71 high, 1.21 wide; ‘neck’ 0.71 wide; bearing two pairs of rudimentary horns; highest point of pars cephalica (HPC) near posterior third of ‘head’ (ratio of HPC to post-ocular length 0.63), carapace gently sloping posterior to HPC; ‘head’ not strongly elevated dorsally (post-ocular ratio 0.26). Chelicerae without accessory setae on anterior face of paturon. Abdomen 1.54 long, 1.37 wide; with four pairs of dorsal hump-like tubercles (HT 1-4). Internal genitalia (Fig. 7G) with cluster of 4-5 variably-shaped spermathecae on either side of gonopore, clusters widely separated along midline of genital plate; innermost (anterior) spermathecae longest, sausage-shaped, bent laterally; other spermathecae variably sausage-shaped or pyriform; posterior pair of spermathecae slightly separated posteriorly.

**Figure 5.**
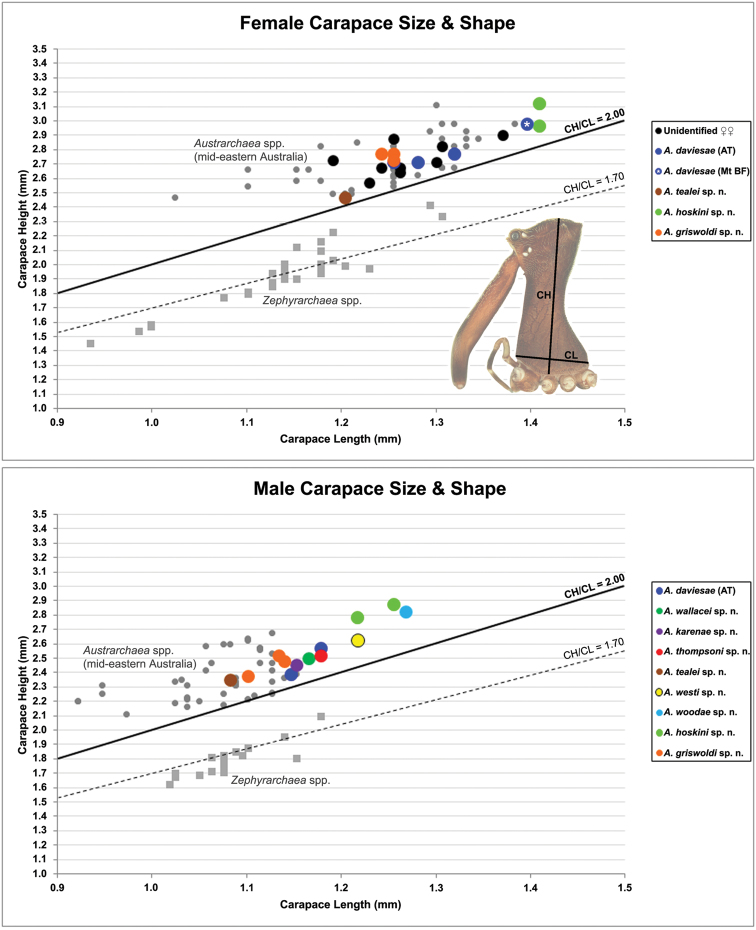
Graphs depicting the relationship between carapace length (CL) and carapace height (CH) for species of *Austrarchaea* from north-eastern Queensland. Smaller grey dots denote species of *Austrarchaea* from mid-eastern Australia (see Rix and Harvey 2011); smaller grey squares denote species of *Zephyrarchaea* from southern Australia (see Rix and Harvey 2012a). Note the relatively large body sizes of *Austrarchaea hoskini* sp. n. and *Austrarchaea woodae* sp. n., and the body size variation between populations of *Austrarchaea daviesae* from the Atherton Tableland (AT) and Mount Bartle Frere (Mt BF), respectively.

*Variation*: Males (Atherton Tableland; n = 2): total length 2.74–3.23; carapace length 1.15–1.18; carapace height 2.38–2.56; CH/CL ratio 2.08–2.17. Females (Atherton Tableland; n = 3): total length 3.44–3.49; carapace length 1.26–1.32; carapace height 2.7–-2.77; CH/CL ratio 2.10–2.15. Females (Mount Bartle Frere; n = 2): total length 3.64–3.79; carapace length 1.40 (invariable); carapace height 2.97 (invariable); CH/CL ratio 2.13 (invariable). Although female specimens from Mount Bartle Frere appear to be slightly larger than those from further west (Fig. 5), carapace proportions and genitalia seem otherwise very similar to specimens from the Atherton Tableland (see Remarks, below).

##### Distribution and habitat.

*Austrarchaea daviesae* is known from the ‘Misty Mountains’ region of the southern Atherton Tableland, in the vicinity of Ravenshoe and Millaa Millaa, with additional specimens also known from Mount Bartle Frere in the adjacent Wooroonooran National Park (see Remarks, below) (Figs 16, 25). Specimens have been collected in pitfall and flight intercept traps, by beating vegetation, or by beating and sifting elevated leaf litter at the bases of lawyer vine palms (*Calamus* spp.) in dense tropical rainforest (Fig. 1F).

##### Conservation status.

This species has a relatively widespread distribution in several National Parks protected under World Heritage legislation, and is not considered to be of conservation concern.

##### Remarks.

The identification and distribution of *Austrarchaea daviesae* has, until recently, been difficult to ascertain, as the holotype male (QMB S1091; Fig. 7B) is without pedipalps (these presumably having been mounted on SEM stubs as per Forster and Platnick 1984, figs 70–74). Similarly, no adult male specimens had been collected from the Atherton Tableland since the original holotype collection in 1978. Fortunately, an adult male and female were collected in early 2012, from the paratype locality (Malaan National Park), near the type locality of Majors Mountain. These specimens (WAM T125183), described above, closely conform to original descriptions, and the male pedipalp appears indistinguishable from that illustrated in Forster and Platnick (1984, figs 70–74). Interestingly, the distribution of *Austrarchaea daviesae* appears to extend beyond the Atherton Tableland, with eastern populations apparently sympatric or at least partly sympatric with *Austrarchaea woodae* sp. n. on Mount Bartle Frere, in the Wooroonooran National Park. Adult Mount Bartle Frere specimens collected by the California Academy of Sciences in 2009 are conspecific with specimens from Malaan National Park, as confirmed by pedipalp images supplied by H. Wood (pers. comm.). Another juvenile specimen from Mount Bartle Frere (CASENT 9023672), collected in 2006, is also conspecific with these adult Mount Bartle Frere specimens, as determined by almost identical *COI* sequences (H. Wood, pers. comm.). Interestingly, female specimens collected by the QM from Boulder Caves, near the type locality of *Austrarchaea woodae* sp. n., also appear to be *Austrarchaea daviesae* (rather than *Austrarchaea woodae* sp. n.), due to the presence of fully developed (rather than recumbent) abdominal tubercles, and a similar carapace morphology and similar genitalia to specimens from the Atherton Tableland. *Austrarchaea woodae* sp. n. thus appears to be much rarer than *Austrarchaea daviesae* at altitudes ≤ ~1000 m, and may actually be restricted to higher altitude montane rainforest on the summit of Mount Bartle Frere.

#### Austrarchaea wallacei

Rix & Harvey sp. n.

urn:lsid:zoobank.org:act:0368CE32-E6E8-4D49-A0C0-1FD3DEFCAC23

http://species-id.net/wiki/Austrarchaea_wallacei

Figs 8 17 25

##### Vernacular name.

Mount Misery Assassin Spider

##### Type material.

Holotype male: Mount Misery, summit, [Monkhouse Timber Reserve], Queensland, Australia, 15°52'S, 145°14'E, pitfall trap, 850 m, 6.XII.1990–17.I.1991, Queensland Museum & ANZSES (QMB S25964).

##### Etymology.

The specific epithet is a patronym in honour of the late Doug Wallace OAM (1923–2012), for his passion and enthusiasm for arachnology, for his contributions to the study of Australian (and especially Queensland) spiders, for his efforts in founding and fostering the Rockhampton Arachnological Society, and for his encouragement of MGR over many years.

##### Diagnosis.

*Austrarchaea wallacei* can be distinguished from all other Archaeidae from north-eastern Queensland except *Austrarchaea karenae* sp. n., *Austrarchaea tealei* sp. n. and *Austrarchaea thompsoni* sp. n. by the presence of a triangular spur on the embolus (Fig. 8D); from *Austrarchaea thompsoni* sp. n. by the presence of a prominent, triangular tegular sclerite 1 (TS 1) (Fig. 8D); and from *Austrarchaea karenae* sp. n.and *Austrarchaea tealei* sp. n. by the shape of tegular sclerite 3 (TS 3), which has a bluntly pointed, triangular apex (Figs 8C–D).

##### Description.

**Figure 6.**
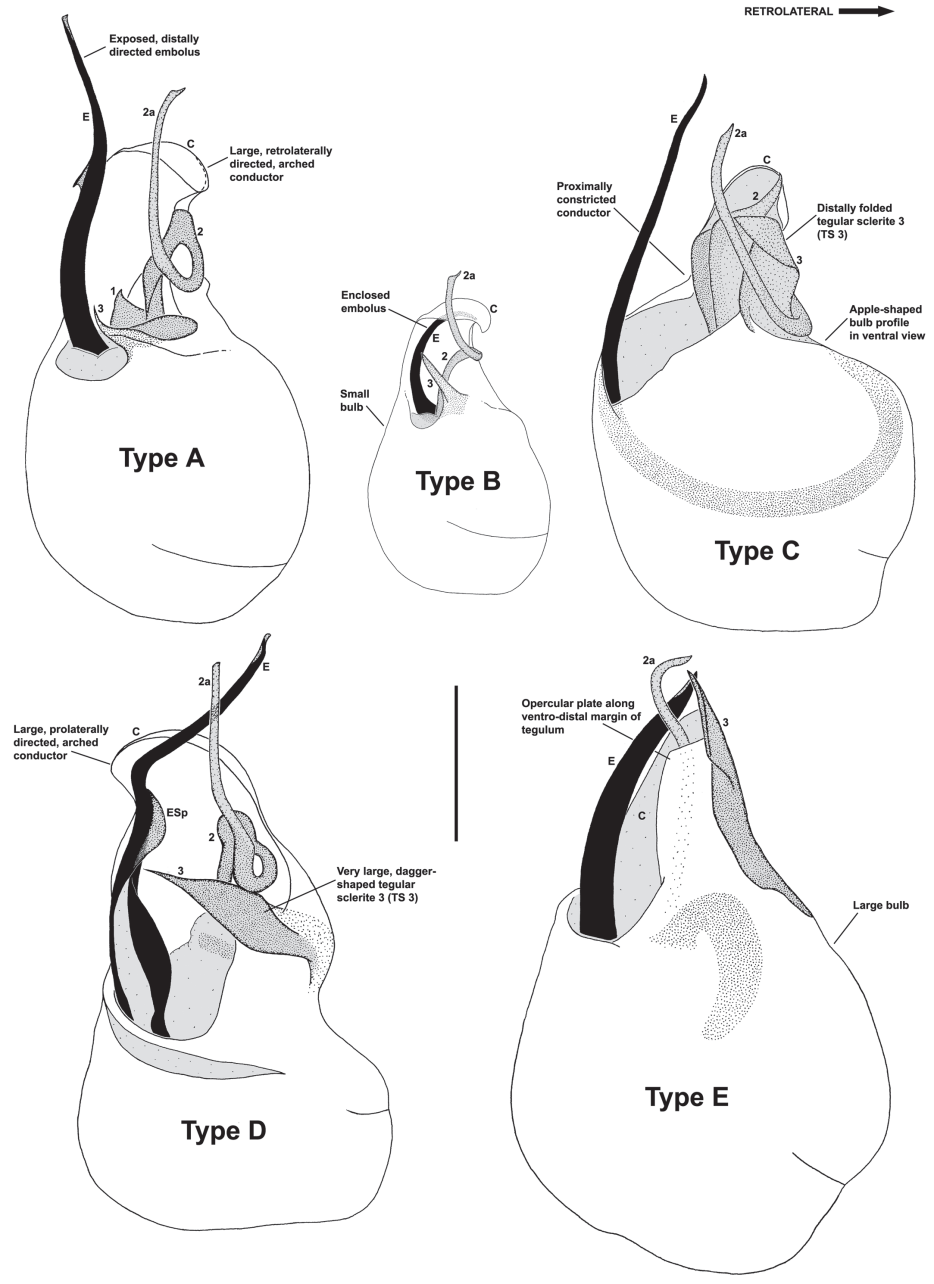
Morphological differences between the five pedipalp types (Types A-E) identified for species of *Austrarchaea* from north-eastern Queensland, with left bulbs illustrated in ventral view at scale-identical sizes. Type A pedipalps are shared among at least five species from the Wet Tropics bioregion; Types B-E are autapomorphic for single species. Note especially the variation in the size and shape of the bulb, and the shape and orientation of the conductor. C = conductor; E = embolus; ESp = embolic spur; (TS)1-3 = tegular sclerites 1-3. Scale bar = 0.2 mm.

**Figure 7.**
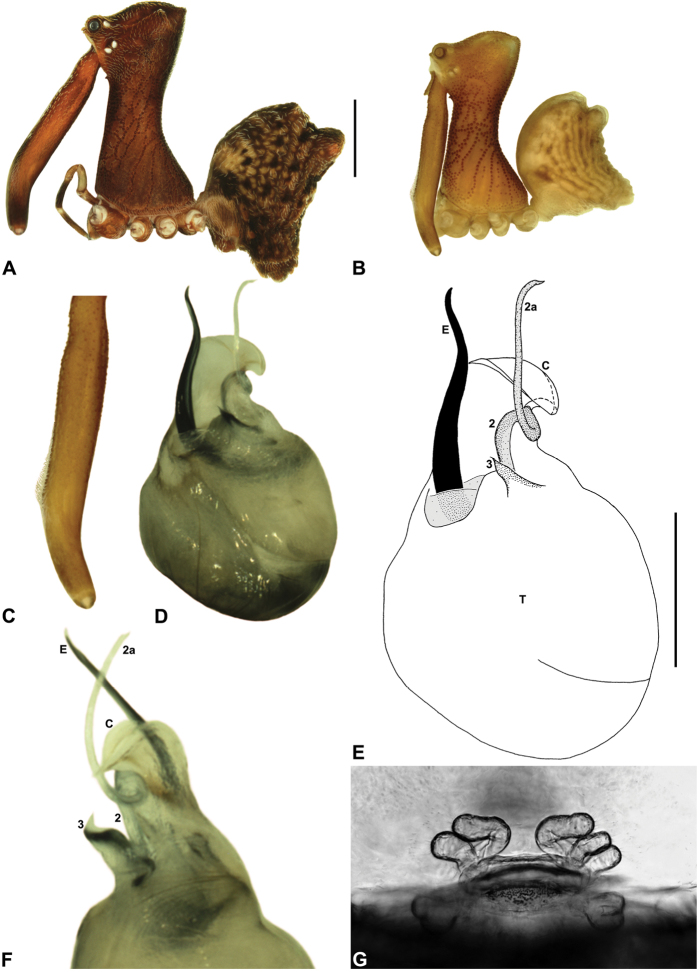
*Austrarchaea daviesae* Forster & Platnick, 1984. **A–B**, Cephalothorax and abdomen, lateral view: **A**, female (WAM T125183) from Malaan National Park, Atherton Tableland, NE. Queensland; **B**, holotype male (QMB S1091) from Majors Mountain, Atherton Tableland, NE. Queensland. **C**, Holotype male chelicerae, lateral view, showing accessory setae. **D–F**, Male (WAM T125183; from Malaan National Park, Atherton Tableland, NE. Queensland) pedipalp: **D–E**, bulb, ventral view; **F**, detail of distal tegular sclerites, retrolateral view. **G**, Female (WAM T125183) internal genitalia, postero-ventral view (genital plate removed). C = conductor; E = embolus; T = tegulum; (TS)2-3 = tegular sclerites 2-3. Scale bars: A-B = 1.0 mm; E = 0.2 mm.

**Figure 8.**
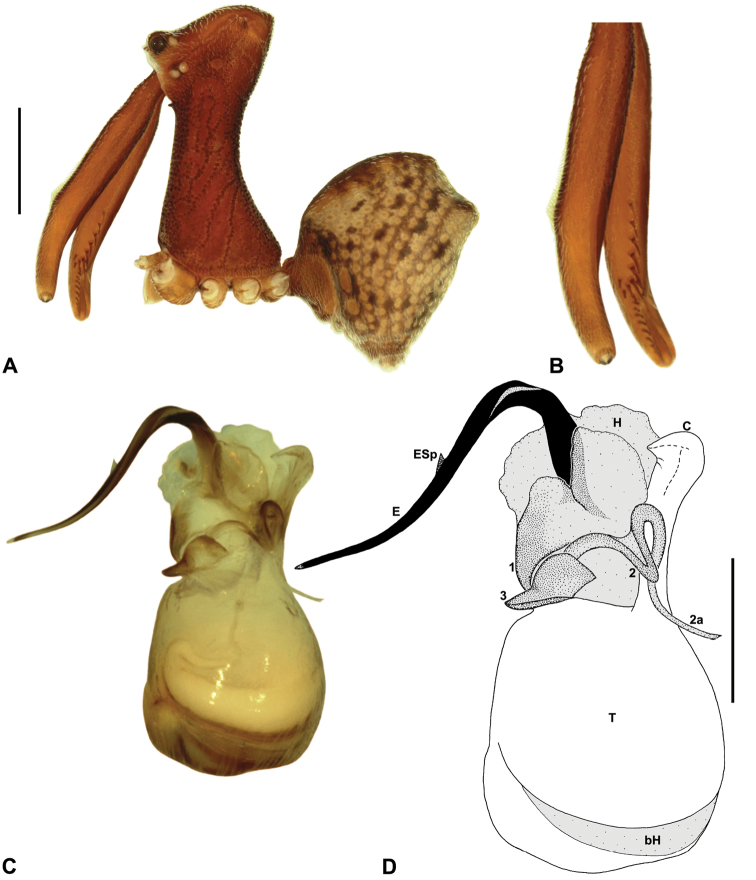
*Austrarchaea wallacei* sp. n. **A–D**, Holotype male (QMB S25964) from Mount Misery, Monkhouse Timber Reserve, NE. Queensland: **A**, cephalothorax and abdomen, lateral view; **B**, chelicerae, lateral view, showing accessory setae; **C–D**, right pedipalpal bulb (expanded; flipped horizontal for inter-specific comparison), retrolateral view. bH = basal haematodocha; C = conductor; E = embolus; ESp = embolic spur; H = haematodocha; T = tegulum; (TS)1-3 = tegular sclerites 1-3. Scale bars: A = 1.0 mm; D = 0.2 mm.

*Holotype male*: Total length 3.28; leg I femur 3.01; F1/CL ratio 2.58. Cephalothorax dark reddish-brown; legs tan-brown with darker annulations; abdomen mottled grey-brown and beige, with darker brown dorsal scute and sclerites (Fig. 8A). Carapace tall (CH/CL ratio 2.14); 1.17 long, 2.49 high, 1.10 wide, ‘neck’ 0.62 wide; bearing two pairs of rudimentary horns; highest point of pars cephalica (HPC) approaching posterior quarter of ‘head’ (ratio of HPC to post-ocular length 0.72), carapace gently sloping posterior to HPC; ‘head’ moderately elevated dorsally (post-ocular ratio 0.33). Chelicerae with short brush of accessory setae on anterior face of paturon (Fig. 8B). Abdomen 1.59 long, 1.28 wide; with two pairs of dorsal hump-like tubercles (HT 1-4); dorsal scute fused anteriorly to epigastric sclerites, extending posteriorly to first pair of hump-like tubercles; HT 3-4 each covered by separate dorsal sclerites. Expanded pedipalp (Figs 8C–D) of Type A morphology (Fig. 6), with large, retrolaterally directed, arched conductor; embolus sinuous, with short triangular spur; tegular sclerite 3 (TS 3) short, spur-like, with flattened proximal portion and bluntly pointed, triangular apex; TS 2-2a flexed dorsally (due to haematodochal expansion), TS 2 with pointed apex; TS 1 triangular, with tapered, slightly curved tooth-like apex.

*Female*: Unknown.

##### Distribution and habitat.

*Austrarchaea wallacei* is known only from the summit of Mount Misery, 34 km north-west of Cape Tribulation (Figs 17, 25). The single known specimen was collected in a pitfall trap in tropical rainforest at 850 m elevation.

##### Conservation status.

Unknown (data deficient).

#### Austrarchaea karenae

Rix & Harvey sp. n.

urn:lsid:zoobank.org:act:E010DAB8-9909-432E-BAA9-0452A1EBCCE0

http://species-id.net/wiki/Austrarchaea_karenae

Figs 9 18 25

##### Vernacular name.

Windsor Tableland Assassin Spider

##### Type material.

Holotype male: Windsor Tableland, [Windsor Tableland National Park], 1.2 km past barracks, Queensland, Australia, 16°15'S, 145°02'E, QM berlesate, stick brushing, rainforest, 1060 m, 24.XI.1997, G. Monteith (QMB S43060).

##### Etymology.

The specific epithet is a patronym in honour of Dr Karen Edward, for her contributions to our understanding of Wet Tropics biogeography, and for her great friendship to MGR and MSH over many years.

##### Diagnosis.

**Figure 9.**
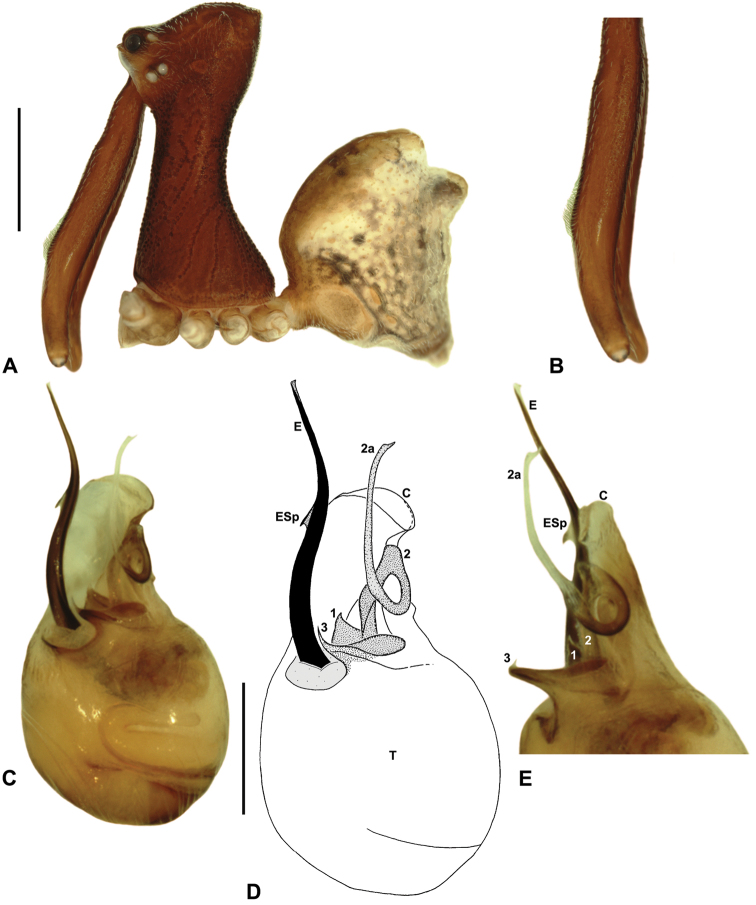
*Austrarchaea karenae* sp. n. **A–E**, Holotype male (QMB S43060) from Windsor Tableland, Windsor Tableland National Park, NE. Queensland: **A**, cephalothorax and abdomen, lateral view; **B**, chelicerae, lateral view, showing accessory setae; **C–D**, pedipalpal bulb, ventral view; **E**, detail of distal tegular sclerites, retrolateral view. C = conductor; E = embolus; ESp = embolic spur; T = tegulum; (TS)1-3 = tegular sclerites 1-3. Scale bars: A = 1.0 mm; D = 0.2 mm.

*Austrarchaea karenae* can be distinguished from all other Archaeidae from north-eastern Queensland except *Austrarchaea tealei* sp. n., *Austrarchaea thompsoni* sp. n. and *Austrarchaea wallacei* by the presence of a triangular spur on the embolus (Figs 9D–E); from *Austrarchaea thompsoni* sp. n. by the presence of a prominent, triangular tegular sclerite 1 (TS 1), which is visible in ventral view (Fig. 9D); and from *Austrarchaea tealei* sp. n. and *Austrarchaea wallacei* by the shape of tegular sclerite 3 (TS 3), which has a single, sharply pointed process distally (Fig. 9D).

##### Description.

*Holotype male*: Total length 2.97; leg I femur 3.17; F1/CL ratio 2.74. Cephalothorax dark reddish-brown; legs tan-brown with darker annulations; abdomen mottled grey-brown and beige, with darker brown dorsal scute and sclerites (Fig. 9A). Carapace tall (CH/CL ratio 2.12); 1.15 long, 2.49 high, 1.09 wide, ‘neck’ 0.61 wide; bearing two pairs of rudimentary horns; highest point of pars cephalica (HPC) near posterior third of ‘head’ (ratio of HPC to post-ocular length 0.65), carapace gently sloping posterior to HPC; ‘head’ moderately elevated dorsally (post-ocular ratio 0.32). Chelicerae with short brush of accessory setae on anterior face of paturon (Fig. 9B). Abdomen 1.59 long, 1.13 wide; with two pairs of dorsal hump-like tubercles (HT 1-4); dorsal scute fused anteriorly to epigastric sclerites, extending posteriorly to first pair of hump-like tubercles; HT 3-4 each covered by separate dorsal sclerites. Unexpanded pedipalp (Figs 9C–E) of Type A morphology (Fig. 6), with large, retrolaterally directed, arched conductor; embolus distally directed, slightly sinuous, with short triangular spur adjacent to distal rim of conductor, embolus projecting beyond distal rim of conductor by ~1/2 length of exposed embolic portion; tegular sclerite 3 (TS 3) short, spur-like, with flattened proximal portion and sharply pointed, claw-like apex; TS 2-2a looped over retrolateral edge of conductor, TS 2 with pointed, subtriangular apex, TS 2a projecting beyond distal rim of conductor but not extending to near tip of embolus; TS 1 broadly triangular in ventral view.

*Female*: Unknown.

##### Distribution and habitat.

*Austrarchaea karenae* is known only from the Windsor Tableland, 44 km north-west of Mossman (Figs 18, 25). The single known specimen was collected in high altitude tropical rainforest.

##### Conservation status.

Unknown (data deficient).

#### Austrarchaea thompsoni

Rix & Harvey sp. n.

urn:lsid:zoobank.org:act:CC84B06D-AD54-41A7-8237-F2031D57F0A7

http://species-id.net/wiki/Austrarchaea_thompsoni

Figs 10 19 25

##### Vernacular name.

Carbine Tableland Assassin Spider

##### Type material.

Holotype male: Devils Thumb area, [Daintree National Park (Mossman Gorge Section)], 10 km NW. of Mossman, Queensland, Australia, [16°27'S, 145°17'E], pyrethrum knockdown, tropical rainforest, 1000–1180 m, 10.X.1982, G. Monteith, D. Yeates, G. Thompson (QMB S30840).

##### Etymology

**.** The specific epithet is a patronym in honour of Geoff Thompson, for his ongoing efforts in collecting and documenting the invertebrate rainforest fauna of the Wet Tropics, and for collecting the only known specimen of this species.

##### Diagnosis.

**Figure 10.**
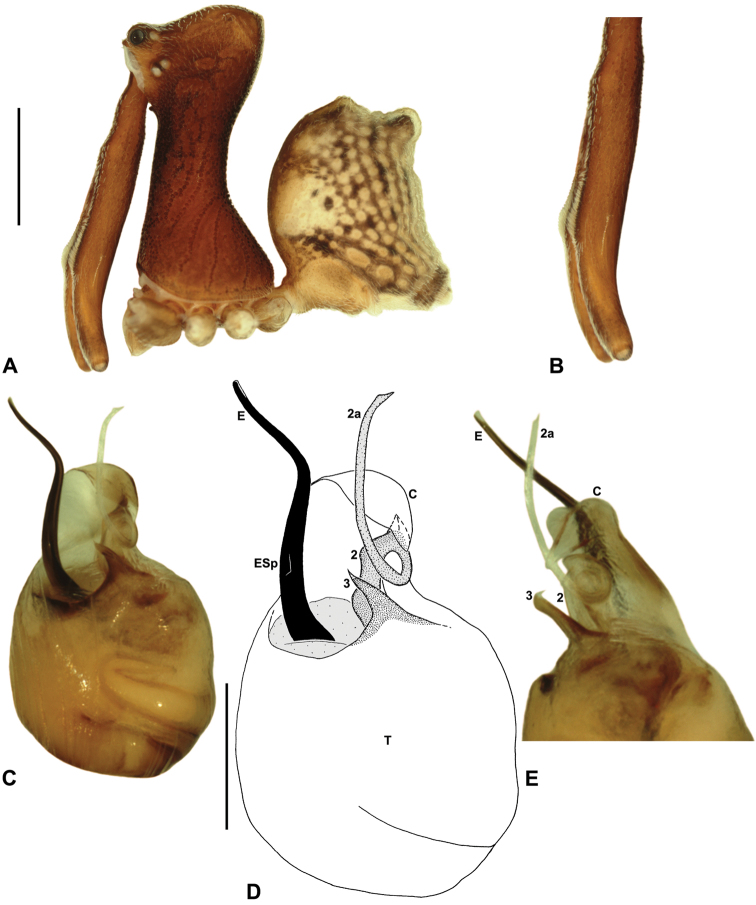
*Austrarchaea thompsoni* sp. n. **A–E**, Holotype male (QMB S30840) from Devils Thumb, Daintree National Park, NE. Queensland: **A**, cephalothorax and abdomen, lateral view; **B**, chelicerae, lateral view, showing accessory setae; **C–D**, pedipalpal bulb, ventral view; **E**, detail of distal tegular sclerites, retrolateral view. C = conductor; E = embolus; ESp = embolic spur; T = tegulum; (TS)2-3 = tegular sclerites 2-3. Scale bars: A = 1.0 mm; D = 0.2 mm.

*Austrarchaea thompsoni* can be distinguished from all other Archaeidae from north-eastern Queensland except *Austrarchaea karenae*, *Austrarchaea tealei* sp. n. and *Austrarchaea wallacei* by the presence of a triangular spur on the embolus (Fig. 10D); and from *Austrarchaea karenae*, *Austrarchaea tealei* sp. n. and *Austrarchaea wallacei* by the very small tegular sclerite 1 (TS 1), which is not visible in ventral view (Fig. 10D), and by the more proximally positioned embolic spur, which is situated near the base of the exposed embolic portion (Fig. 10D).

##### Description.

*Holotype male*: Total length 2.97; leg I femur 3.23; F1/CL ratio 2.74. Cephalothorax dark reddish-brown; legs tan-brown with darker annulations; abdomen mottled grey-brown and beige, with darker brown dorsal scute and sclerites (Fig. 10A). Carapace tall (CH/CL ratio 2.13); 1.18 long, 2.51 high, 1.13 wide, ‘neck’ 0.63 wide; bearing two pairs of rudimentary horns; highest point of pars cephalica (HPC) near posterior third of ‘head’ (ratio of HPC to post-ocular length 0.69), carapace gently sloping posterior to HPC; ‘head’ not strongly elevated dorsally (post-ocular ratio 0.25). Chelicerae with short brush of accessory setae on anterior face of paturon (Fig. 10B). Abdomen 1.64 long, 1.23 wide; with two pairs of dorsal hump-like tubercles (HT 1-4); dorsal scute fused anteriorly to epigastric sclerites, extending posteriorly to first pair of hump-like tubercles; HT 3-4 each covered by separate dorsal sclerites. Unexpanded pedipalp (Figs 10C–E) of Type A morphology (Fig. 6), with large, retrolaterally directed, arched conductor; embolus distally directed, slightly sinuous, with short triangular spur situated near base of exposed embolic portion, embolus projecting beyond distal rim of conductor by slightly less than 1/2 length of exposed embolic portion; tegular sclerite 3 (TS 3) short, spur-like, with constricted tegular base and sharply pointed, claw-like apex; TS 2-2a looped beneath overhanging retrolateral edge of conductor, TS 2 with rounded, subtriangular apex, TS 2a projecting beyond distal rim of conductor to near tip of embolus; TS 1 very small, obscured by TS 2-3, not visible in ventral view.

*Female*: Unknown.

##### Distribution and habitat.

*Austrarchaea thompsoni* is known only from Devils Thumb, on the Carbine Tableland 10 km west-north-west of Mossman (Figs 19, 25). The single known specimen was collected in high altitude tropical rainforest.

##### Conservation status.

Unknown (data deficient).

#### Austrarchaea tealei

Rix & Harvey sp. n.

urn:lsid:zoobank.org:act:60D9ADD6-1BDB-4964-B484-99587C772CAB

http://species-id.net/wiki/Austrarchaea_tealei

Figs 11 20 25

Austrarchaea sp. n. ‘(NEQ-1)’ Rix and Harvey 2012b: 379, figs 3, 5–7.

##### Vernacular name.

Mossman Gorge Assassin Spider

##### Type material.

Holotype male: Daintree National Park (Mossman Gorge Section), Mossman Gorge, off water access road ~50 m from carpark, Queensland, Australia, 16°28'20"S, 145°19'53"E, sifting elevated leaf litter under lawyer vine palms, tropical rainforest, 78 m, 21.III.2012, M. & A. Rix (QMB S92210).

##### Other material examined.

**AUSTRALIA: *Queensland*: Daintree National Park (Mossman Gorge Section):** Mossman Gorge, [16°28'20"S, 145°19'53"E], 23.IV.1967, D. Colless, 1♀ (ANIC); Mossman Gorge, Water Access Track, Site 2, 16°28'28"S, 145°19'41"E, sieved litter from around roots and rocks on shady steep section of bank, tropical rainforest, 1.IV.2009, K. Edward, J. Waldock, 1 juvenile (WAM T97462).

##### Other material (not examined).

**AUSTRALIA: *Queensland*: Daintree National Park (Mossman Gorge Section):** Mossman Gorge, carpark, 16°28'20"S, 145°19'52"E, day collecting, turning over logs, rainforest, 45 m, 17–18.IV.2009, H. Wood, 4♂, 2♀ (CASENT 9028385).

##### Etymology.

The specific epithet is a patronym in honour of Roy Teale, for his friendship to MSH, for his efforts in facilitating systematic research at the Western Australian Museum, and for his crucial support of the Western Australian Museum’s ‘archaeid project’ since 2007.

##### Diagnosis.

**Figure 11.**
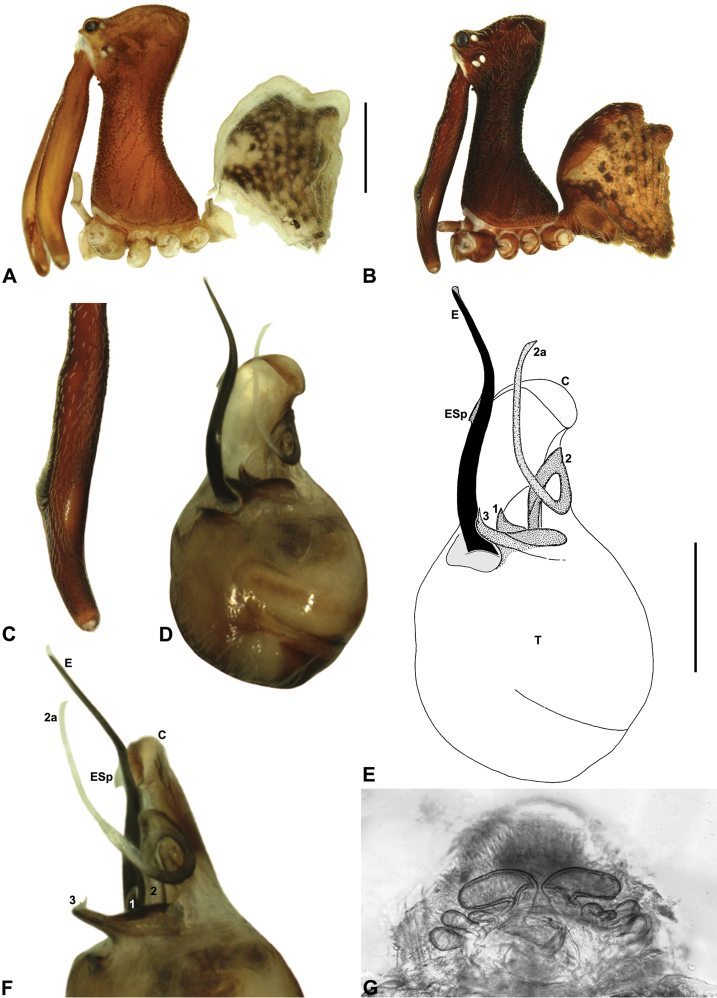
*Austrarchaea tealei* sp. n. **A–B**, Cephalothorax and abdomen, lateral view: **A**, female (ANIC) from Mossman Gorge, Daintree National Park, NE. Queensland; **B**, holotype male (QMB S92210) from Mossman Gorge, Daintree National Park, NE. Queensland. **C**, Holotype male chelicerae, lateral view, showing accessory setae. **D–F**, Holotype male pedipalp: **D–E**, bulb, ventral view; **F**, detail of distal tegular sclerites, retrolateral view. **G**, Female (ANIC) internal genitalia, postero-ventral view (genital plate removed). C = conductor; E = embolus; ESp = embolic spur; T = tegulum; (TS)1-3 = tegular sclerites 1-3. Scale bars: A-B = 1.0 mm; E = 0.2 mm.

*Austrarchaea tealei* can be distinguished from all other Archaeidae from north-eastern Queensland except *Austrarchaea karenae*, *Austrarchaea thompsoni* and *Austrarchaea wallacei* by the presence of a triangular spur on the embolus (Figs 11E–F); from *Austrarchaea thompsoni* by the presence of a prominent, triangular tegular sclerite 1 (TS 1), which is visible in ventral view (Fig. 11E); and from *Austrarchaea karenae* and *Austrarchaea wallacei* by the shape of tegular sclerite 3 (TS 3), which has a second short, pointed process distally (Fig. 11E).

##### Description.

*Holotype male*: Total length 2.67; leg I femur 3.09; F1/CL ratio 2.85. Cephalothorax dark reddish-brown; legs tan-brown with darker annulations; abdomen mottled dark grey-brown and beige, with darker brown dorsal scute and sclerites (Fig. 11B). Carapace tall (CH/CL ratio 2.17); 1.08 long, 2.35 high, 1.00 wide, ‘neck’ 0.54 wide; bearing two pairs of rudimentary horns; highest point of pars cephalica (HPC) approaching posterior quarter of ‘head’ (ratio of HPC to post-ocular length 0.71), carapace gently sloping posterior to HPC; ‘head’ moderately elevated dorsally (post-ocular ratio 0.30). Chelicerae with short brush of accessory setae on anterior face of paturon (Fig. 11C). Abdomen 1.44 long, 1.05 wide; with two pairs of dorsal hump-like tubercles (HT 1-4); dorsal scute fused anteriorly to epigastric sclerites, extending posteriorly to first pair of hump-like tubercles; HT 3-4 each covered by separate dorsal sclerites. Unexpanded pedipalp (Figs 11D–F) of Type A morphology (Fig. 6), with large, retrolaterally directed, arched conductor; embolus distally directed, slightly sinuous, with short triangular spur adjacent to distal rim of conductor, embolus projecting beyond distal rim of conductor by ~1/2 length of exposed embolic portion; tegular sclerite 3 (TS 3) short, spur-like, with flattened proximal portion and sharply pointed, claw-like apex bearing second short, pointed process distally; TS 2-2a looped over retrolateral edge of conductor, TS 2 with rounded, subtriangular apex, TS 2a projecting beyond distal rim of conductor but not extending to near tip of embolus; TS 1 triangular, with tapered, slightly curved tooth-like apex.

*Female* (ANIC): Total length 2.95; leg I femur 2.86; F1/CL ratio 2.37. Cephalothorax reddish-brown; legs pale tan-brown with darker annulations; abdomen mottled grey-brown and beige (Fig. 11A). Carapace tall (CH/CL ratio 2.04); 1.21 long, 2.46 high, 1.13 wide; ‘neck’ 0.69 wide; bearing two pairs of rudimentary horns; highest point of pars cephalica (HPC) near posterior third of ‘head’ (ratio of HPC to post-ocular length 0.65), carapace gently sloping posterior to HPC; ‘head’ not strongly elevated dorsally (post-ocular ratio 0.23). Chelicerae without accessory setae on anterior face of paturon. Abdomen 1.64 long, 1.28 wide; with four pairs of dorsal hump-like tubercles (HT 1-4). Internal genitalia (Fig. 11G) with cluster of 4-6 variably-shaped spermathecae on either side of gonopore, clusters widely separated along midline of genital plate; innermost (anterior) spermathecae longest, sausage-shaped, bent laterally; other spermathecae variably sausage-shaped or pyriform, smallest anteriorly, becoming progressively larger posteriorly.

##### Distribution and habitat.

*Austrarchaea tealei* is known only from Mossman Gorge, 4.5 km west-south-west of Mossman (Figs 20, 25). Specimens have been collected under logs (as newly-hatched juveniles; H. Wood, pers. comm.), or by beating and sifting elevated leaf litter at the bases of lawyer vine palms (*Calamus* spp.) in lowland tropical rainforest.

##### Conservation status.

Unknown (data deficient).

##### Remarks.

The female specimen described above (from the ANIC) is tentatively identified as conspecific with the holotype of *Austrarchaea tealei*, despite a somewhat different carapace morphology and a fairly imprecise collection locality. *Austrarchaea thompsoni* does occur on nearby mountains above the Mossman River, and thus it possible (albeit unlikely) that the female specimen from “Mossman Gorge” (collected in 1967) may actually belong to another species. We have described it here in the absence of evidence suggesting any sympatry in the Mossman Gorge region, given the fact that all other recently collected Mossman Gorge material appears to be conspecific with the holotype (including CAS material; H. Wood, pers. comm.), and given the similarly small body size of this female specimen and the holotype male (Fig. 5).

#### Austrarchaea westi

Rix & Harvey sp. n.

urn:lsid:zoobank.org:act:1D560E1E-96FB-4F5B-A826-281429ADD500

http://species-id.net/wiki/Austrarchaea_westi

Figs 12 21 25

##### Vernacular name.

Lamb Range Assassin Spider

##### Type material.

Holotype male: Mount Williams, [Dinden National Park], 16°55'S, 145°40'E, pyrethrum, trees and logs, 1000 m, 2.XII.1993, G. Monteith, H. Janetzki (QMB S59537).

**Other material examined. AUSTRALIA: *Queensland*: Dinden National Park:** same data as holotype, 1 juvenile (QMB S59537).

##### Etymology.

The specific epithet is a patronym in honour of Paul West, for his friendship to MSH over many years, and for helping fund the Western Australian Museum’s ‘archaeid project’ from 2009–2012.

##### Diagnosis.

**Figure 12.**
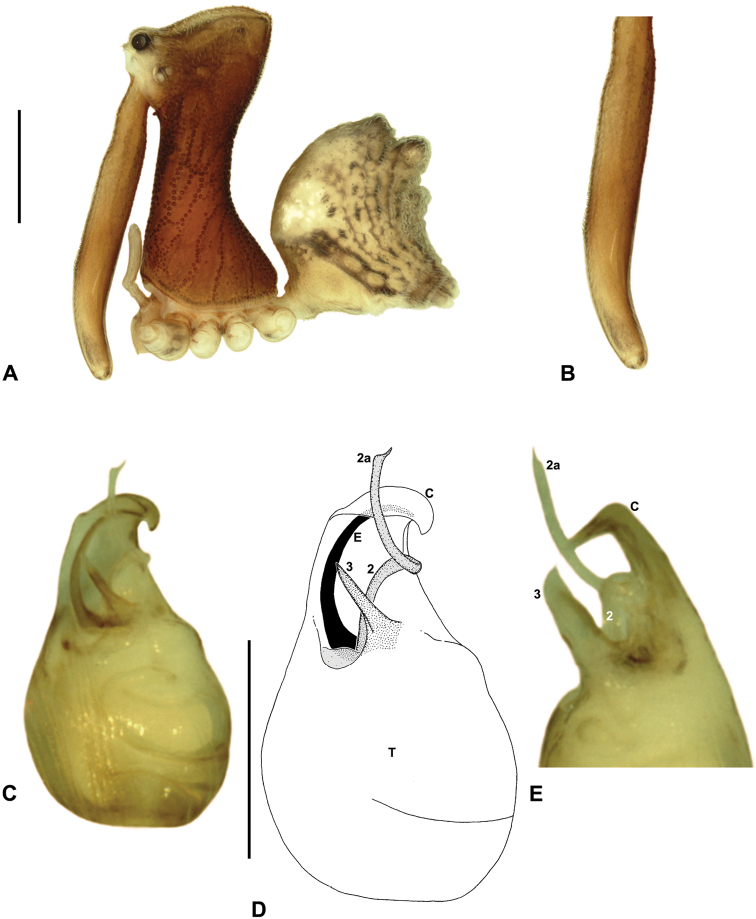
*Austrarchaea westi* sp. n. **A–E**, Holotype male (QMB S59537) from Mount Williams, Dinden National Park, NE. Queensland: **A**, cephalothorax and abdomen, lateral view; **B**, chelicerae, lateral view, showing lack of defined accessory setae; **C–D**, pedipalpal bulb, ventral view; **E**, detail of distal tegular sclerites, retrolateral view. C = conductor; E = embolus; T = tegulum; (TS)2-3 = tegular sclerites 2-3. Scale bars: A = 1.0 mm; D = 0.2 mm.

*Austrarchaea westi* can be distinguished from all other Archaeidae from north-eastern Queensland by the presence of a unique Type B pedipalp (Fig. 6), with very small bulb (width << 0.30 mm) (Figs 6, 12D), and by the relatively short embolus, which is distally enclosed within the conductor (Figs 6, 12D). This species can be further distinguished by the very short, barely differentiated accessory setae on the male chelicerae (Fig. 12B).

##### Description.

*Holotype male*: Total length 3.13; leg I femur 3.23; F1/CL ratio 2.65. Cephalothorax reddish-brown; legs beige with darker annulations; abdomen mottled grey-brown and beige, with darker brown dorsal scute and sclerites (Fig. 12A). Carapace tall (CH/CL ratio 2.15); 1.22 long, 2.62 high, 1.13 wide, ‘neck’ 0.65 wide; bearing two pairs of rudimentary horns; highest point of pars cephalica (HPC) approaching posterior quarter of ‘head’ (ratio of HPC to post-ocular length 0.71), carapace gently sloping posterior to HPC; ‘head’ not strongly elevated dorsally (post-ocular ratio 0.27). Chelicerae with very short, barely differentiated accessory setae on anterior face of paturon (Fig. 12B). Abdomen 1.65 long, 1.10 wide; with two pairs of dorsal hump-like tubercles (HT 1-4); dorsal scute fused anteriorly to epigastric sclerites, extending posteriorly to first pair of hump-like tubercles; HT 3-4 each covered by separate dorsal sclerites. Unexpanded pedipalp (Figs 12C-E) of Type B morphology (Fig. 6), very small in size (width of bulb << 0.30), with large, retrolaterally directed, arched conductor; embolus curved, distally enclosed within conductor, without spur; tegular sclerite 3 (TS 3) porrect, spur-like, with pointed, pro-distally directed apex; TS 2-2a looped over retrolateral edge of conductor, TS 2 not strongly developed distally, TS 2a projecting beyond distal rim of conductor; TS 1 very small, obscured by TS 2-3, not visible in ventral view.

*Female*: Unknown.

##### Distribution and habitat.

*Austrarchaea westi* is known only from Mount Williams, on the Lamb Range 11 km west of Cairns (Figs 21, 25). The two known specimens were collected in high altitude tropical rainforest.

##### Conservation status.

Unknown (data deficient).

#### Austrarchaea woodae

Rix & Harvey sp. n.

urn:lsid:zoobank.org:act:E4F1A9F7-33E3-4FCF-86CE-C14A1572F037

http://species-id.net/wiki/Austrarchaea_woodae

Figs 13 22 25

##### Vernacular name.

Mount Bartle Frere Assassin Spider

##### Type material.

Holotype male: Mount Bartle Frere, [Wooroonooran National Park], Boulder Caves, Queensland, Australia, [17°23'S, 145°47'E], 1050 m, 8.XII.1990, G. Monteith, G. Thompson, D. Cook, R. Sheridan (QMB S72988).

##### Etymology.

The specific epithet is a patronym in honour of Dr Hannah Wood, for her pioneering research into the systematics, biology and biogeography of assassin spiders and other Palpimanoidea, and for her collaborative support of MGR and MSH during assassin spider research conducted at the Western Australian Museum.

##### Diagnosis.

**Figure 13.**
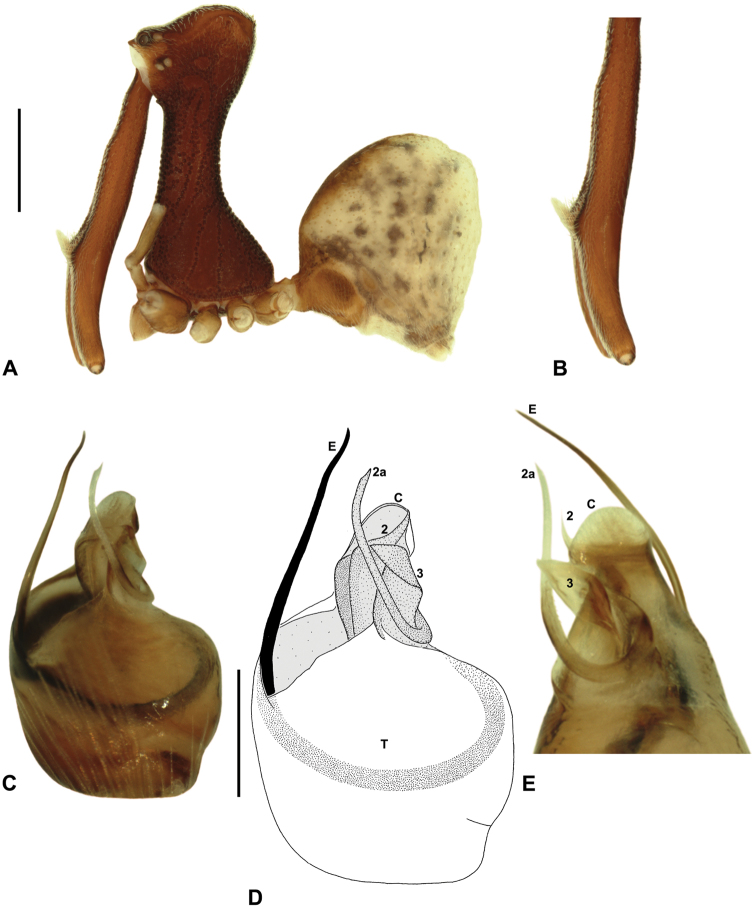
*Austrarchaea woodae* sp. n. **A–E**, Holotype male (QMB S72988) from Boulder Caves, Mount Bartle Frere, Wooroonooran National Park, NE. Queensland: **A**, cephalothorax and abdomen, lateral view; **B**, chelicerae, lateral view, showing accessory setae; **C–D**, pedipalpal bulb, ventral view; **E**, detail of distal tegular sclerites, retrolateral view. C = conductor; E = embolus; T = tegulum; (TS)2-3 = tegular sclerites 2-3. Scale bars: A = 1.0 mm; D = 0.2 mm.

*Austrarchaea woodae* can be distinguished from all other Archaeidae from north-eastern Queensland by the presence of a unique Type C pedipalp (Fig. 6), with a proximally constricted conductor (Figs 6, 13D), large, flattened, distally folded tegular sclerite 3 (TS 3) (Figs 6, 13D–E), and apple-shaped bulb profile in ventral view (Figs 6, 13C–D). This species can be further distinguished by the dense, pick-like tuft of accessory setae on the male chelicerae (Fig. 13B; similar only to *Austrarchaea harmsi* among Australian Archaeidae), and by the almost spherical abdomen with recumbent hump-like tubercles (Fig. 13A; similar only to species of *Zephyrarchaea* among Australian Archaeidae).

##### Description.

*Holotype male*: Total length 3.54; leg I femur 3.74; F1/CL ratio 2.95. Cephalothorax dark reddish-brown; legs tan-brown with darker annulations; abdomen mottled grey-brown and beige, with darker brown dorsal scute and sclerites (Fig. 13A). Carapace very tall (CH/CL ratio 2.22); 1.27 long, 2.82 high, 1.18 wide, ‘neck’ 0.56 wide; bearing two pairs of rudimentary horns; highest point of pars cephalica (HPC) near posterior third of ‘head’ (ratio of HPC to post-ocular length 0.67), carapace steeply sloping and convex posterior to HPC; ‘head’ moderately elevated dorsally (post-ocular ratio 0.31). Chelicerae with dense, pick-like tuft of accessory setae on anterior face of paturon (Fig. 13B). Abdomen 1.69 long, 1.44 wide; almost spherical, with largely recumbent hump-like tubercles; dorsal scute fused anteriorly to epigastric sclerites. Unexpanded pedipalp (Figs 13C–E) of Type C morphology (Fig. 6), with retrolaterally directed, proximally constricted conductor and apple-shaped bulb profile in ventral view; embolus distally directed, slightly sinuous, without spur; tegular sclerite 3 (TS 3) large, flattened, with prominent, distally folded apex; TS 2-2a looped over retrolateral edge of conductor, TS 2 with strongly developed, spur-like apex extending to near distal rim of conductor, TS 2a looping around TS 3 proximally and projecting beyond distal rim of conductor to near tip of embolus; TS 1 indistinct, obscured by TS 2-3.

*Female*: Unknown.

##### Distribution and habitat.

*Austrarchaea woodae* is known only from near the summit of Mount Bartle Frere, 12 km south-west of Babinda (Figs 22, 25). The only known specimen was collected in high altitude tropical rainforest.

##### Conservation status.

Unknown (data deficient).

##### Remarks.

See Remarks for *Austrarchaea daviesae* (above).

#### Austrarchaea hoskini

Rix & Harvey sp. n.

urn:lsid:zoobank.org:act:432C741C-00BA-4E9A-999C-8E0343C9F199

http://species-id.net/wiki/Austrarchaea_hoskini

Figs 14 23 25

Austrarchaea sp. n. Rix and Harvey 2012b: 376, fig. 1G.

##### Vernacular name.

Mount Elliot Assassin Spider

##### Type material.

Holotype male: Mount Elliot, [Bowling Green Bay National Park], Upper North Creek, Queensland, Australia, [19°29'S, 146°57'E], rainforest, 1000 m, 2–5.XII.1986, G. Monteith, G. Thompson, S. Hamlet (QMB S30811).

##### Paratypes.

Allotype female, Mount Elliot, [Bowling Green Bay National Park], North Creek, Queensland, Australia, 19°29'S, 146°57'E, 1000 m, 25–27.III.1991, G. Monteith, D. Cook (QMB S17937); 1 male, 1 female, same data (QMB S23045).

##### Other material examined.

**AUSTRALIA: *Queensland*: Bowling Green Bay National Park:** same data as holotype, 1 juvenile (QMB S30811); same data as holotype except pitfall trap, 3–5.XII.1986, 1 juvenile (QMB S30839).

##### Etymology.

The specific epithet is a patronym in honour of Dr Conrad Hoskin, for his contributions to our understanding of Wet Tropics biogeography, and for his efforts in documenting the remarkable endemic biota of Mount Elliot.

##### Diagnosis.

**Figure 14.**
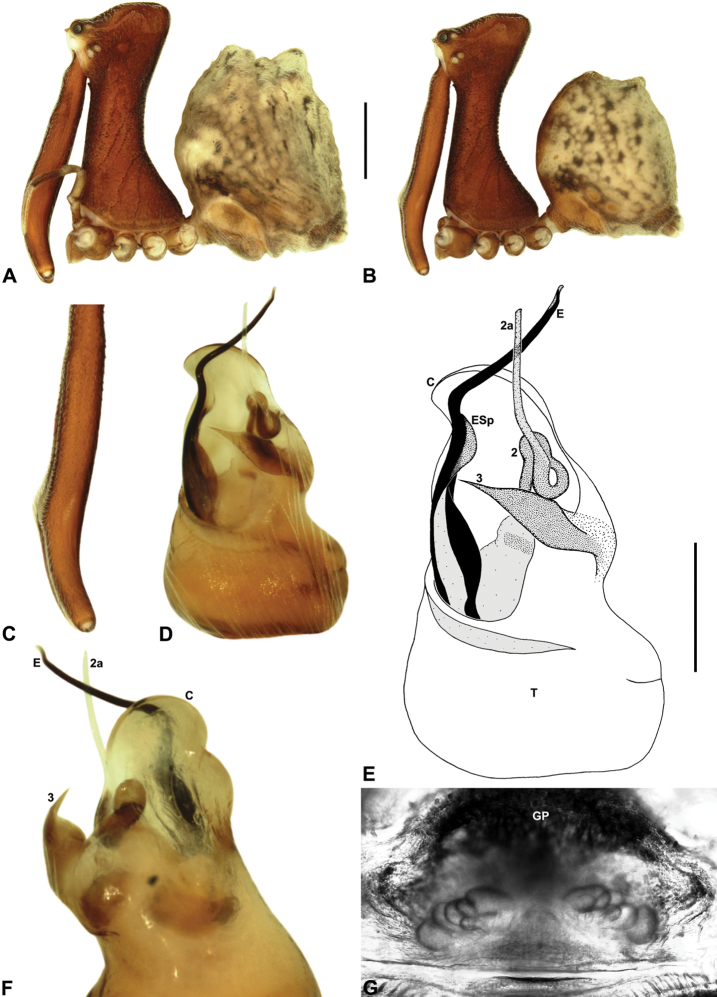
*Austrarchaea hoskini* sp. n. **A–B**, Cephalothorax and abdomen, lateral view: **A**, allotype female (QMB S17937) from Mount Elliot, Bowling Green Bay National Park, NE. Queensland; **B**, holotype male (QMB S30811) from Mount Elliot, Bowling Green Bay National Park, NE. Queensland. **C**, Holotype male chelicerae, lateral view, showing accessory setae. **D–F**, Holotype male pedipalp: **D–E**, bulb, ventral view; **F**, detail of distal tegular sclerites, retrolateral view. **G**, Allotype female internal genitalia, postero-ventral view (as seen through posterior rim of genital plate). C = conductor; E = embolus; ESp = embolic spur; GP = genital plate; T = tegulum; (TS)2-3 = tegular sclerites 2-3. Scale bars: A-B = 1.0 mm; E = 0.2 mm.

*Austrarchaea hoskini* can be distinguished from all other Archaeidae from north-eastern Queensland by the presence of a unique Type D pedipalp (Fig. 6), with a prolaterally directed conductor (Figs 6, 14E), very large, dagger-shaped tegular sclerite 3 (TS 3) (Figs 6, 14E–F), and prominent, rounded, fin-shaped embolic spur (Fig. 14E).

##### Description.

*Holotype male*: Total length 3.44; leg I femur 3.59; F1/CL ratio 2.86. Cephalothorax dark reddish-brown; legs tan-brown with darker annulations; abdomen mottled grey-brown and beige, with darker brown dorsal scute and sclerites (Fig. 14B). Carapace very tall (CH/CL ratio 2.29); 1.26 long, 2.87 high, 1.21 wide, ‘neck’ 0.59 wide; bearing two pairs of rudimentary horns; highest point of pars cephalica (HPC) near posterior third of ‘head’ (ratio of HPC to post-ocular length 0.63), carapace almost horizontal posterior to HPC; ‘head’ not strongly elevated dorsally (post-ocular ratio 0.28). Chelicerae with short brush of accessory setae on anterior face of paturon (Fig. 14C). Abdomen 2.00 long, 1.54 wide; with two pairs of dorsal hump-like tubercles (HT 1-4); dorsal scute fused anteriorly to epigastric sclerites, extending posteriorly to first pair of hump-like tubercles; HT 3-4 each covered by separate dorsal sclerites. Unexpanded pedipalp (Figs 14D–F) of Type D morphology (Fig. 6), with large, prolaterally directed, arched conductor; embolus distally directed, sinuous, with prominent, rounded, fin-shaped spur proximal to distal kink in embolus, embolus projecting beyond distal rim of conductor by ~1/3 length of exposed embolic portion; tegular sclerite 3 (TS 3) very large, dagger-like, directed pro-ventrally across bulb; TS 2-2a forming looped, figure-of-eight-shaped structure in ventral view, TS 2 rounded distally, TS 2a projecting beyond distal rim of conductor to near tip of embolus; TS 1 very small, indistinct, probably embedded within haematodochal membranes.

*Allotype female*: Total length 3.79; leg I femur 3.44; F1/CL ratio 2.44. Cephalothorax dark reddish-brown; legs tan-brown with darker annulations; abdomen mottled grey-brown and beige (Fig. 14A). Carapace tall (CH/CL ratio 2.10); 1.41 long, 2.96 high, 1.31 wide; ‘neck’ 0.72 wide; bearing two pairs of rudimentary horns; highest point of pars cephalica (HPC) approaching posterior quarter of ‘head’ (ratio of HPC to post-ocular length 0.71), carapace almost horizontal anterior and slightly posterior to HPC; ‘head’ not strongly elevated dorsally (post-ocular ratio 0.29). Chelicerae without accessory setae on anterior face of paturon. Abdomen 2.31 long, 1.87 wide; with four pairs of dorsal hump-like tubercles (HT 1-4). Internal genitalia (Fig. 14G) with cluster of ~6 variably-shaped spermathecae on either side of gonopore, clusters widely separated along midline of genital plate; innermost (anterior) spermathecae longest, sausage-shaped, bent laterally; other spermathecae variably sausage-shaped or pyriform.

*Variation*: Males (n = 2): total length 3.38–3.44; carapace length 1.22–1.26; carapace height 2.78–2.87; CH/CL ratio 2.28–2.29. Females (n = 2): total length 3.59–3.79; carapace length 1.41 (invariable); carapace height 2.96–3.12; CH/CL ratio 2.10–2.21.

##### Distribution and habitat.

*Austrarchaea hoskini* is known only from Mount Elliot, 30 km south-east of Townsville (Figs 23, 25). The few known specimens were collected in high altitude rainforest along North Creek.

##### Conservation status.

Unknown (data deficient).

#### Austrarchaea spp. (unidentified specimens)

##### Note.

In the absence of adult male specimens or molecular data, the following female and juvenile specimens (see Figs 16–23, 25) could not be confidently identified as known species. Species of *Austrarchaea* are difficult to identify (and diagnose) by females alone, and in the Wet Tropics these difficulties were compounded by the absence of representative adults from across the region. Material is thus here listed according to upland subregional zones of faunal endemism, as proposed for the Wet Tropics bioregion (see Discussion, below; Table 1; Figs 16B–23B).

**Table 1.** List of upland subregional zones of faunal endemism identified for the Wet Tropics bioregion (by Winter et al. 1984, Williams et al. 1996 and other authors) (see Discussion; Figs 16B-23B), noting current collection records of Archaeidae, including the presence of any described species. Subregional zones are listed from the northern-most Mt Finnigan Uplands (Fig. 16B) to the southern-most Elliot Uplands (Fig. 23B). Note the addition of Hinchinbrook Island (as per Edward 2011), and the current absence of archaeid collections from four of the southern subregions. F = female specimen/s; J = juvenile specimen/s; M = male specimen/s; N.R. = no recorded specimens.

**Wet Tropics Upland Subregion**	**Archaeidae**	**Described species**
Mt Finnigan Uplands (FU)	Yes^M,F,J^	*Austrarchaea wallacei*
Thornton Uplands (TU)	Yes^F,J^	
Windsor Uplands (WU)	Yes^M^	*Austrarchaea karenae*
Carbine Uplands (CU)	Yes^M,F,J^	*Austrarchaea tealei*, *Austrarchaea thompsoni*
Black Mountain Corridor (BM)	Yes^J^	
Lamb Uplands (LU)	Yes^M,J^	*Austrarchaea westi*
Malbon-Thompson Uplands (MT)	Yes^F,J^	
Bellenden Ker/Bartle Frere (BK)	Yes^M,F,J^	*Austrarchaea daviesae*, *Austrarchaea woodae*
Atherton Uplands (AU)	Yes^M,F,J^	*Austrarchaea daviesae*
Kirrama Uplands (KU)	Yes^J^	
Hinchinbrook Island (HI)	N.R.	
Lee Uplands (LE)	N.R.	
Spec Uplands (SU)	N.R.	
Halifax Uplands (HU)	N.R.	
Elliot Uplands (EU)	Yes^M,F,J^	*Austrarchaea hoskini*

##### Material examined.

**AUSTRALIA: *Queensland*:** **FINNIGAN UPLANDS: Monkhouse Timber Reserve:** Moses Creek, 4 km NNE. of Mount Finnigan, 15°47'S, 145°17'E, berlesate, sieved rainforest litter, 14–16.X.1980, T. Weir, 1♀ (ANIC); Mount Boolbun South, 15°57'S, 145°08'E, 850–1000 m, 4–6.XI.1995, G. Monteith, D. Cook, L. Roberts, 1♀ (QMB S41070). **Cedar Bay National Park:** Mount Hartley, 15°47'S, 145°19'E, pyrethrum, trees & logs, 750 m, 8.XI.1995, G. Monteith, 1 juvenile (QMB). **THORNTON UPLANDS: Daintree National Park (Cape Tribulation Section):** Mount Sorrow Ridge Walk, centre saddle ~1.5 km from start, 16°04'35"S, 145°27'32"E, sifting elevated leaf litter under lawyer vine palms, tropical rainforest, 203 m, 20.III.2012, M. & A. Rix, 2♀, 1 juvenile (WAM T125629); same data, 1♀ (QMB S92211); on track to Mount Sorrow, 16°04'43"S, 145°27'42"E, day collecting, sifting leaf litter, mini-winklers, rainforest, 600 m, 20.IV.2009, H. Wood, 1♀ (CASENT 9028390); 4 km W. of Cape Tribulation (Site 8), 16°05'S, 145°26'E, QM berlesate, stick brushing, rainforest, 720 m, 29–30.IX.1982, G. Monteith, D. Yeates, G. Thompson, 1 juvenile (QMB S30802); 5 km W. of Cape Tribulation (Site 10), pyrethrum knockdown, rainforest, 780 m, 28.IX.1982, G. Monteith, D. Yeates, G. Thompson, 1 juvenile (QMB S30818); 4.5–5 km W. of Cape Tribulation (Top Camp), pyrethrum knockdown, rainforest, 760–780 m, 1–6.X.1982, G. Monteith, D. Yeates, G. Thompson, 1 juvenile (QMB S30825); Thornton Peak, via Daintree, 1100–1300 m, 24–27.IX.1984, G. & S. Monteith, 1 juvenile (QMB S30801). **Monkhouse Timber Reserve:** Mount Pieter Botte, 16°04'S, 145°24'E, pyrethrum, trees, logs, rocks, 950 m, 21.XI.1983, G. Monteith, H. Janetzki, 1 juvenile (QMB). **CARBINE UPLANDS: Daintree National Park (Mossman Gorge Section):** Upper Whyanbeel Creek, 16°23'S, 145°17'E, pyrethrum, mossy rocks, 1150 m, 5.IX.1992, G. Monteith, 3 juveniles (QMB S38582). **Mount Lewis Forest Reserve:** Mount Lewis, summit, via Julatten, QM berlesate, stick brushings, rainforest, 1200 m, 10.IX.1981, G. Monteith, D. Cook, 1 juvenile (QMB S30841). **Mount Spurgeon Forest Reserve:** Mount Spurgeon, summit, 16°26'S, 145°12'E, 1320 m, 21.XI.1997, G. Monteith, D. Cook, C. Burwell, 1♀ (QMB S35869). **BLACK MOUNTAIN CORRIDOR: Mowbray National Park:** Black Mountain, 17 km ESE. of Julatten, pyrethrum knockdown, 800–1000 m, 29–30.IV.1982, G. Monteith, D. Yeates, D. Cook, 1 juvenile (QMB S30813). **LAMB UPLANDS: Danbulla National Park:** Mount Haig, 17°06'S, 145°36'E, pitfall trap, 1150 m, 4–31.V.1995, P. Zborowski, 1 juvenile (ANIC). **Dinden National Park:** Isley Hills, 17°03'S, 145°42'E, pyrethrum, trees and rocks, 1050 m, 30.XI.1993, G. Monteith, H. Janetzki, 1 juvenile (QMB S59692). **MALBON-THOMPSON UPLANDS: Russell River National Park:** Graham Range, 17°17'S, 145°58'E, pyrethrum, logs, 550 m, 8–9.XII.1995, G. Monteith, G. Thompson, D. Cook, 1♀ (QMB S37969); same data except pyrethrum, trees and logs, 1.XI.1995, G. Monteith, 2 juveniles (QMB). **BELLENDEN KER/BARTLE FRERE: Wooroonooran National Park:** Bellenden Ker Range, 0.5 km south of Cable Tower No. 7, pyrethrum knockdown on logs, stones and tree trunks, 500 m, 17–24.X.1981, Earthwatch, QM, 1 juvenile (QMB S30828); Massey Range, 17°16'S, 145°49'E, QM berlesate, sieved litter, rainforest, 1250 m, 10.X.1991, G. Monteith, H. Janetzki, 1 juvenile (QMB S49636). **ATHERTON UPLANDS: Herberton Range National Park:** Longlands Gap, 17°28'S, 145°29'E, pitfall trap, 1150 m, 4.II.–6.III.1995, P. Zborowski, 1 juvenile (ANIC). **Herberton Range State Forest:** Baldy Mountain Road, 7 km SW. Atherton, pyrethrum, logs & trees, 1150 m, 9.XII.1988, G. Monteith, G. Thompson, 1 juvenile (QMB). **Tully Gorge National Park:** Mount Tyson, 2 km W. of Tully, 17°55'S, 145°54'E, QM berlesate, sieved litter, rainforest, 650 m, 7.V.1983, D. Yeates, 1 juvenile (QMB S30800); Upper Boulder Creek, via Tully, 17°50'S, 145°54'E, QM berlesate, sieved litter, rainforest, 900 m, 27.X.1983, G. Monteith, D. Yeates, G. Thompson, 1 juvenile (QMB S30805); Upper Boulder Creek, 10 km N. of Tully, 800 m, 4–5.XII.1989, G. Monteith, G. Thompson, H. Janetzki, 1♀ (QMB S73924); Upper Boulder Creek, 11 km NNW. of Tully, 850 m, 16–19.XI.1984, D. Cook, G. Monteith, G. Thompson, 1♀ (QMB S30815); same data except pyrethrum, logs & trees, 1000 m, 5.XII.1989, G. Monteith, G. Thompson, H. Janetzki, 1 juvenile (QMB). **Wooroonooran National Park:** Hughes Road, Topaz, 17°26'S, 145°42'E, pitfall trap, 650 m, VII.–IX.1993, G. Monteith, S. Breeden, 1 juvenile (QMB S25715); “Palmerston National Park”, 17°35'30"S, 145°42'00"E, pitfall trap, rainforest, 670 m, 25.VII.–30.XI.1992, R. Raven, P. & E. Lawless, M. Shaw, 1 juvenile (QMB S21921). **KIRRAMA UPLANDS: Girringun National Park:** Cardwell Range, Upper Broadwater Creek Valley, pitfall trap, rainforest, 750 m, 18.XII.1986–14.I.1987, G. Monteith, G. Thompson, S. Hamlet, 1 juvenile (QMB S30842).

### The Mackay-Whitsundays Hinterland fauna

#### Austrarchaea griswoldi

Rix & Harvey sp. n.

urn:lsid:zoobank.org:act:5E8091DF-D073-49CB-B6AC-9D730BC126A6

http://species-id.net/wiki/Austrarchaea_griswoldi

**Figure 15.**
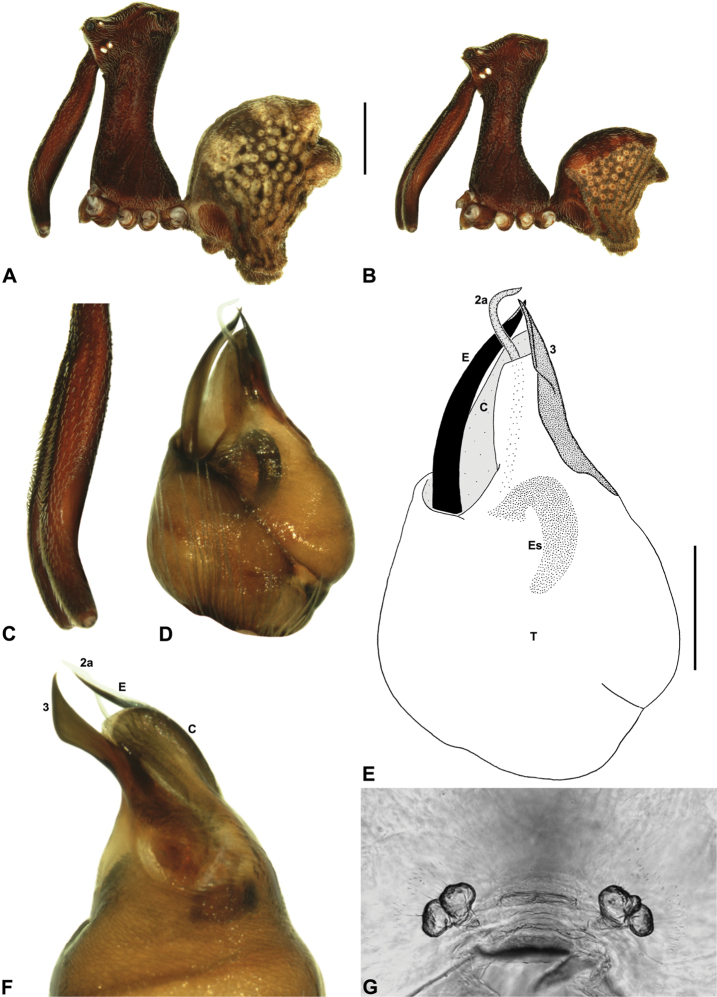
*Austrarchaea griswoldi* sp. n. **A–B**, Cephalothorax and abdomen, lateral view: **A**, allotype female (QMB S92213) from Broken River, Eungella National Park, NE. Queensland; **B**, holotype male (QMB S92212) from Broken River, Eungella National Park, NE. Queensland. **C**, Holotype male chelicerae, lateral view, showing accessory setae. **D–F**, Holotype male pedipalp: **D–E**, bulb, ventral view; **F**, detail of distal tegular sclerites, retrolateral view. **G**, Allotype female internal genitalia, postero-ventral view (genital plate removed). C = conductor; E = embolus; Es = embolic sclerite; T = tegulum; (TS)1-3 = tegular sclerites 1-3. Scale bars: A-B = 1.0 mm; E = 0.2 mm.

Figs 1A–D , 15 24 –25

Austrarchaea sp. n. ‘(NEQ-2)’ Rix and Harvey 2012b: 379, figs 3, 6.

##### Vernacular name.

Eungella Assassin Spider

##### Type material.

Holotype male: Eungella National Park (Broken River Section), Broken River Rainforest Discovery Circuit and Granite Bend Circuit, Queensland, Australia, 21°10'07"S, 148°30'22"E, sifting elevated leaf litter under palms (especially fan palms), tropical rainforest, 684 m, 23.III.2012, M. & A. Rix (QMB S92212).

Paratypes: Allotype female, same data as holotype (QMB S92213); 2 males, 1 female and 2 juveniles, same data as holotype (WAM T125630); 1 female, same data as holotype except Broken River Rainforest Discovery Circuit, hand collecting at night, 24.III.2012 (QMB S92214).

##### Other material examined.

**AUSTRALIA: *Queensland*: Eungella National Park:** Broken River Rainforest Walk, 21°10'02"S, 148°30'23"E, litter, night collection, 720 m, 30.XI.2008, H. Smith, 1 juvenile (AMS KS106561); off Crediton Road Loop, 21°11'09"S, 148°31'43"E, sifting elevated leaf litter under fan palms, tropical rainforest, 673 m, 24.III.2012, M. & A. Rix, 1 juvenile (WAM T125631). **Eungella:** Schoolhouse rainforest general collection, 21°08'S, 148°29'E, 11–15.II.1986, R. Raven, J. Gallon, 1 juvenile (QMB S7039).

##### Etymology.

The specific epithet is a patronym in honour of Dr Charles Griswold, for his outstanding contributions to arachnology, and for his contributions to the study of Archaeidae and other basal Araneomorphae.

##### Diagnosis.

*Austrarchaea griswoldi* can be distinguished from all other Archaeidae from north-eastern Queensland by the presence of a unique Type E pedipalp (Fig. 6), with a very large bulb (width >> 0.30 mm) (Figs 6, 15E), modified ventro-distal rim of the tegulum forming rectangular opercular plate (Figs 6, 15E), and very large, flattened tegular sclerite 3 (TS 3), the latter extending along the entire retrolateral edge of the conductor (Fig. 15F). This species can be further distinguished by the very short, barely differentiated comb of accessory setae on the male chelicerae (Fig. 15C), and by the presence of only two pairs of female spermathecae (Fig. 15G).

##### Description.

*Holotype male*: Total length 3.08; leg I femur 2.99; F1/CL ratio 2.62. Cephalothorax dark reddish-brown; legs dark tan-brown with darker annulations; abdomen mottled dark grey-brown and beige, with darker brown dorsal scute and sclerites (Fig. 15B). Carapace tall (CH/CL ratio 2.17); 1.14 long, 2.47 high, 1.11 wide, ‘neck’ 0.58 wide; bearing two pairs of rudimentary horns; highest point of pars cephalica (HPC) near middle of ‘head’ (ratio of HPC to post-ocular length 0.56), carapace sloping in straight plane posterior to HPC; ‘head’ not strongly elevated dorsally (post-ocular ratio 0.27). Chelicerae with very short, barely differentiated comb of accessory setae on anterior face of paturon (Fig. 15C). Abdomen 1.47 long, 1.05 wide; with two pairs of dorsal hump-like tubercles (HT 1-4); dorsal scute fused anteriorly to epigastric sclerites, extending posteriorly to first pair of hump-like tubercles; HT 3-4 each covered by separate dorsal sclerites. Unexpanded pedipalp (Figs 15D-F) of Type E morphology (Fig. 6), very large in size (width of bulb >> 0.30), with retrolaterally directed, arched conductor; ventro-distal rim of tegulum distally extended to form rectangular opercular plate; embolus distally directed, curved, without spur, projecting only slightly beyond distal rim of conductor; tegular sclerite 3 (TS 3) very large, flattened, extending along entire retrolateral edge of conductor; TS 2-2a largely obscured by rectangular opercular plate, TS 2a projecting beyond distal rim of conductor to just past tip of embolus; TS 1 deeply embedded in bulb, obscured by opercular plate, not visible in ventral view.

*Allotype female*: Total length 3.68; leg I femur 2.87; F1/CL ratio 2.29. Cephalothorax dark reddish-brown; legs tan-brown with darker annulations; abdomen mottled dark grey-brown and beige (Fig. 15A). Carapace tall (CH/CL ratio 2.20); 1.26 long, 2.77 high, 1.21 wide; ‘neck’ 0.70 wide; bearing two pairs of rudimentary horns; highest point of pars cephalica (HPC) near middle of ‘head’ (ratio of HPC to post-ocular length 0.55), carapace sloping in straight plane posterior to HPC; ‘head’ not strongly elevated dorsally (post-ocular ratio 0.28). Chelicerae without accessory setae on anterior face of paturon. Abdomen 1.92 long, 1.59 wide; with four pairs of dorsal hump-like tubercles (HT 1-4). Internal genitalia (Fig. 15G) with pair of pyriform spermathecae on either side of gonopore, clusters widely separated along midline of genital plate.

*Variation*: Males (n = 3): total length 2.87–3.08; carapace length 1.10–1.14; carapace height 2.37–2.51; CH/CL ratio 2.15–2.22. Females (n = 3): total length 3.03–3.68; carapace length 1.24–1.26; carapace height 2.72–2.77; CH/CL ratio 2.16–2.23.

##### Distribution and habitat.

*Austrarchaea griswoldi* is known only from Eungella National Park, 70 km west of Mackay (Figs 24–25). Specimens have been collected by beating and sifting elevated leaf litter in tropical rainforest (Fig. 1E), especially under the dead fronds of Eungella Fan Palms (*Livistona* sp.).

##### Natural history.

A single female specimen was collected by MGR during night collecting in March 2012, suspended with her egg-sac in a tangled maternal web decorated with hanging debris, at the base of a large standing rainforest tree trunk. This egg-sac (Fig. 1D) was carried with both legs IV, positioned behind and against the posterior face of the abdomen, and was composed of soft brown silk. The shape of the egg-sac was irregular, with two large projections, and 18 spiderlings hatched out of the egg-sac on 3-4 April 2012.

##### Conservation status.

This species appears to be a short-range endemic taxon (Harvey 2002b, Harvey et al. 2011), which although potentially restricted in distribution, is abundant within the Eungella National Park (MGR, pers. obs.). It is not considered to be of conservation concern.

#### Austrarchaea spp. (unidentified specimens)

##### Note.

In the absence of adult male specimens or molecular data, the following female specimens (see Figs 24–25) could not be confidently identified as known species.

##### Material examined.

**AUSTRALIA: *Queensland*:** **Dryander National Park:** Mount Dryander, [17 km WNW. of Airlie Beach], pyrethrum, 800 m, 21.XI.1992, G. Monteith, G. Thompson, H. Janetzki, 1♀ (QMB S49380). **Eungella National Park:** Finch Hatton [Gorge], sweeping, complex notophyll vine forest (CNVF), 7–14.IV.1975, R. Kitching, V. Davies, 1♀, 1 juvenile (QMB S1093).

## Discussion

**Figure 16.**
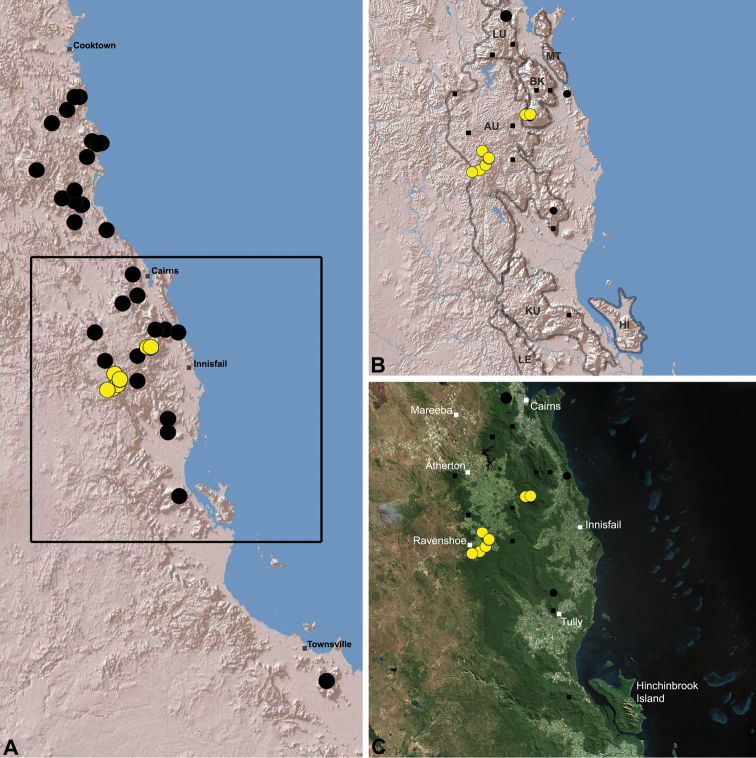
Distribution of *Austrarchaea daviesae* Forster & Platnick, 1984: **A**, topographic map showing the known distribution of Archaeidae in the north-eastern Queensland Wet Tropics bioregion, with collection localities for *Austrarchaea daviesae* highlighted in yellow; **B–C**, topographic and satellite maps showing detail of inset (A). Labelled boundaries in (B) denote upland subregional zones of faunal endemism identified by Winter et al. (1984), Williams et al. (1996) and other authors for the central Wet Tropics (modified from Edward 2011). Small squares in (B–C) denote unidentified juvenile specimens; small circles denote unidentified female specimens; large circles denote described species of *Austrarchaea*. AU = Atherton Uplands; BK = Bellenden Ker/Bartle Frere; HI = Hinchinbrook Island; KU = Kirrama Uplands; LE = Lee Uplands; LU = Lamb Uplands; MT = Malbon-Thompson Uplands.

**Figure 17.**
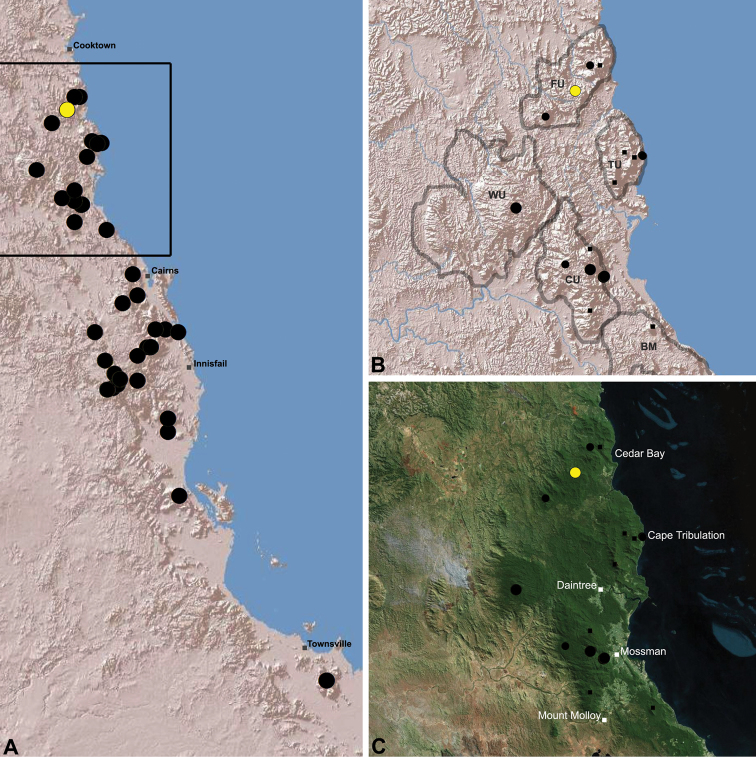
Distribution of *Austrarchaea wallacei* sp. n.: **A**, topographic map showing the known distribution of Archaeidae in the north-eastern Queensland Wet Tropics bioregion, with collection localities for *Austrarchaea wallacei* highlighted in yellow; **B–C**, topographic and satellite maps showing detail of inset (A). Labelled boundaries in (B) denote upland subregional zones of faunal endemism identified by Winter et al. (1984), Williams et al. (1996) and other authors for the northern Wet Tropics (modified from Edward 2011). Small squares in (B–C) denote unidentified juvenile specimens; small circles denote unidentified female specimens; large circles denote described species of *Austrarchaea*. BM = Black Mountain Corridor; CU = Carbine Uplands; FU = Mt Finnigan Uplands; TU = Thornton Uplands; WU = Windsor Uplands.

**Figure 18.**
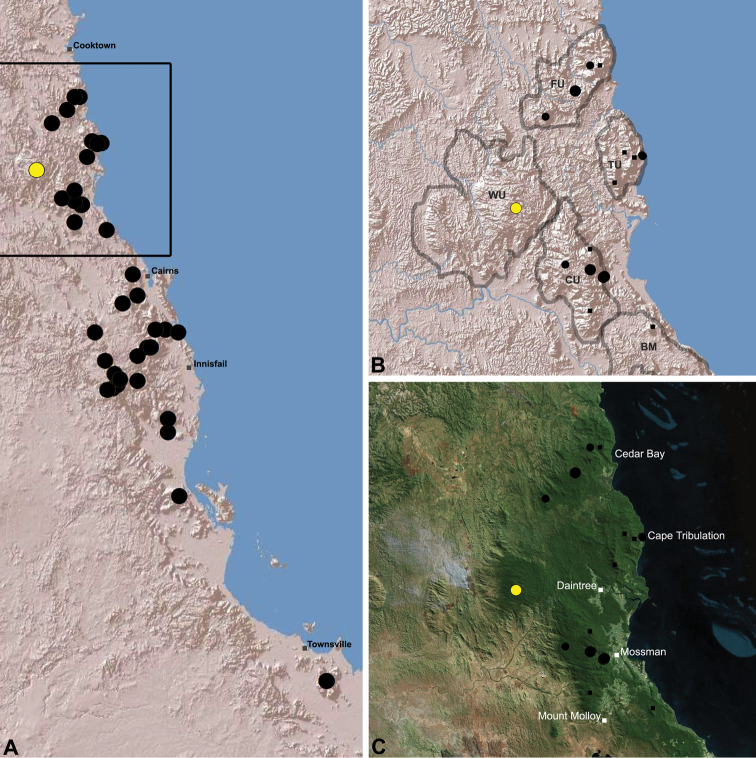
Distribution of *Austrarchaea karenae* sp. n.: **A**, topographic map showing the known distribution of Archaeidae in the north-eastern Queensland Wet Tropics bioregion, with collection localities for *Austrarchaea karenae* highlighted in yellow; **B–C**, topographic and satellite maps showing detail of inset (A). Labelled boundaries in (B) denote upland subregional zones of faunal endemism identified by Winter et al. (1984), Williams et al. (1996) and other authors for the northern Wet Tropics (modified from Edward 2011). Small squares in (B–C) denote unidentified juvenile specimens; small circles denote unidentified female specimens; large circles denote described species of *Austrarchaea*. BM = Black Mountain Corridor; CU = Carbine Uplands; FU = Mt Finnigan Uplands; TU = Thornton Uplands; WU = Windsor Uplands.

**Figure 19.**
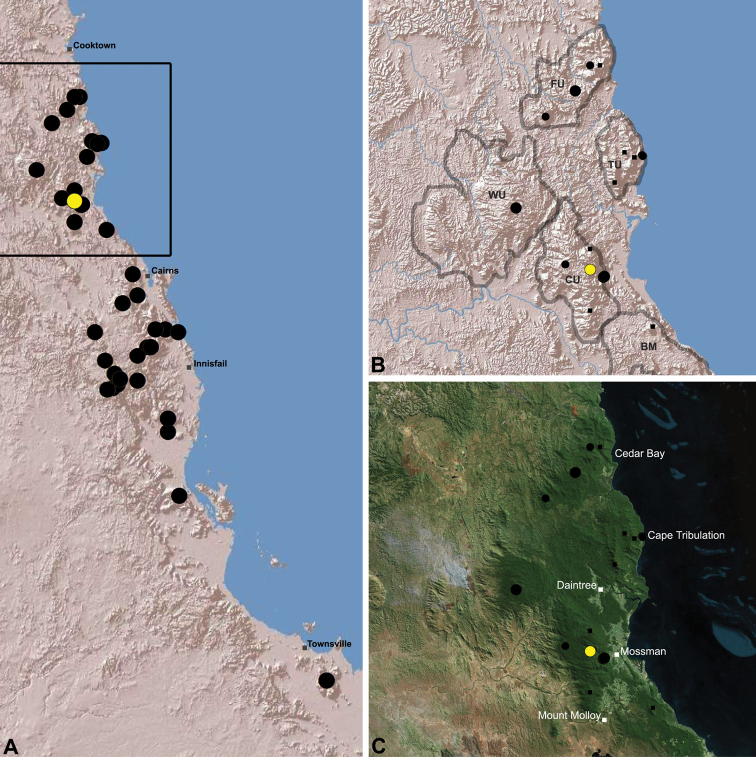
Distribution of *Austrarchaea thompsoni* sp. n.: **A**, topographic map showing the known distrib ution of Archaeidae in the north-eastern Queensland Wet Tropics bioregion, with collection localities for *Austrarchaea thompsoni* highlighted in yellow; **B–C**, topographic and satellite maps showing detail of inset (A). Labelled boundaries in (B) denote upland subregional zones of faunal endemism identified by Winter et al. (1984), Williams et al. (1996) and other authors for the northern Wet Tropics (modified from Edward 2011). Small squares in (B–C) denote unidentified juvenile specimens; small circles denote unidentified female specimens; large circles denote described species of *Austrarchaea*. BM = Black Mountain Corridor; CU = Carbine Uplands; FU = Mt Finnigan Uplands; TU = Thornton Uplands; WU = Windsor Uplands.

**Figure 20.**
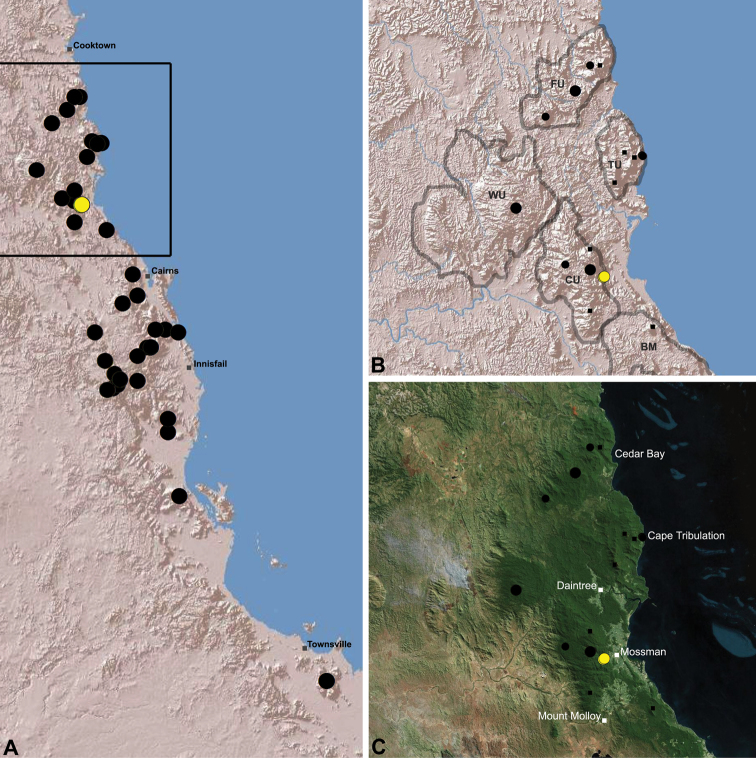
Distribution of *Austrarchaea tealei* sp. n.: **A**, topographic map showing the known distribution of Archaeidae in the north-eastern Queensland Wet Tropics bioregion, with collection localities for *Austrarchaea tealei* highlighted in yellow; **B-C**, topographic and satellite maps showing detail of inset (A). Labelled boundaries in (B) denote upland subregional zones of faunal endemism identified by Winter et al. (1984), Williams et al. (1996) and other authors for the northern Wet Tropics (modified from Edward 2011). Small squares in (B–C) denote unidentified juvenile specimens; small circles denote unidentified female specimens; large circles denote described species of *Austrarchaea*. BM = Black Mountain Corridor; CU = Carbine Uplands; FU = Mt Finnigan Uplands; TU = Thornton Uplands; WU = Windsor Uplands.

**Figure 21.**
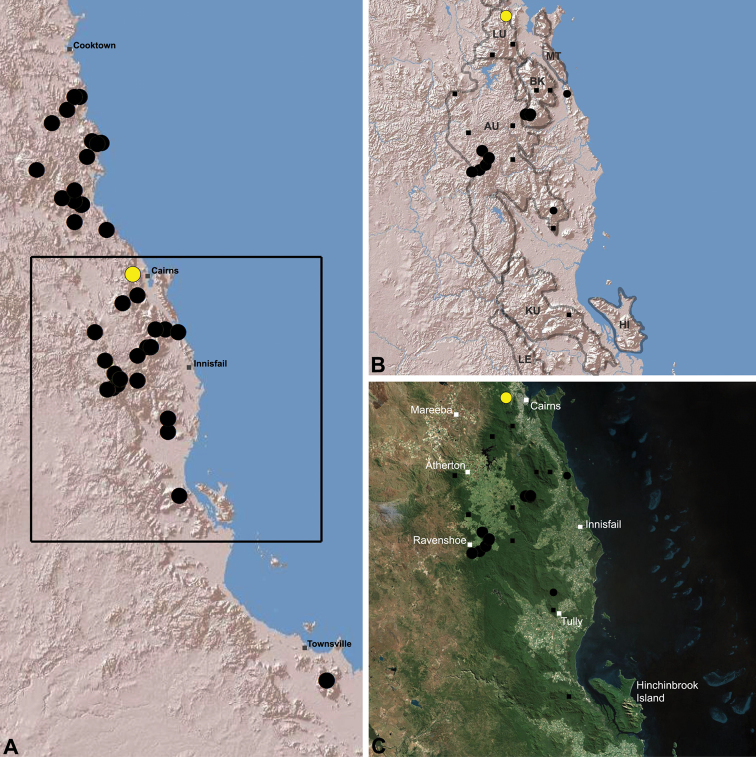
Distribution of *Austrarchaea westi* sp. n.: **A**, topographic map showing the known distribution of Archaeidae in the north-eastern Queensland Wet Tropics bioregion, with collection localities for *Austrarchaea westi* highlighted in yellow; **B-C**, topographic and satellite maps showing detail of inset (A). Labelled boundaries in (B) denote upland subregional zones of faunal endemism identified by Winter et al. (1984), Williams et al. (1996) and other authors for the central Wet Tropics (modified from Edward 2011). Small squares in (B–C) denote unidentified juvenile specimens; small circles denote unidentified female specimens; large circles denote described species of *Austrarchaea*. AU = Atherton Uplands; BK = Bellenden Ker/Bartle Frere; HI = Hinchinbrook Island; KU = Kirrama Uplands; LE = Lee Uplands; LU = Lamb Uplands; MT = Malbon-Thompson Uplands.

**Figure 22.**
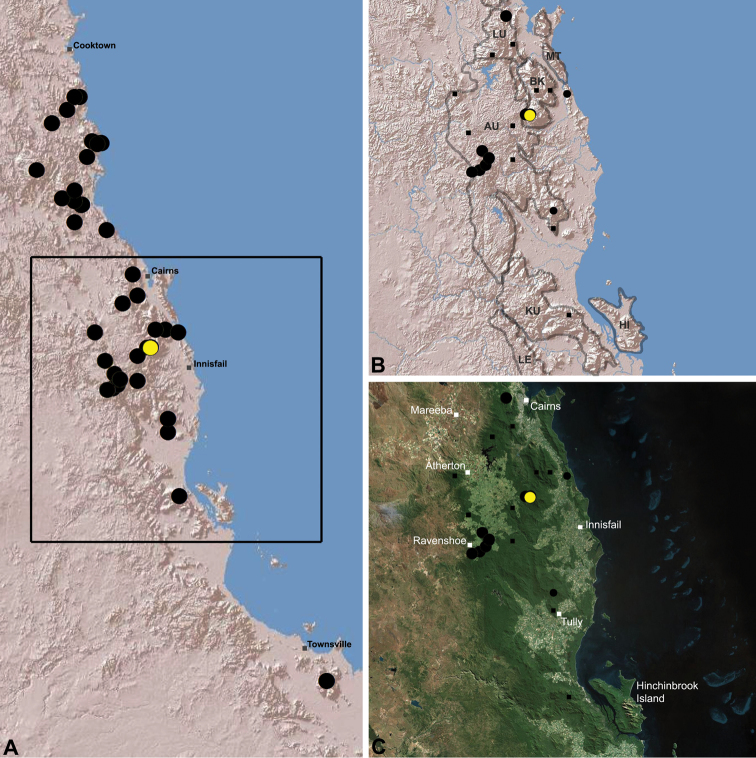
Distribution of *Austrarchaea woodae* sp. n.: **A**, topographic map showing the known distribution of Archaeidae in the north-eastern Queensland Wet Tropics bioregion, with collection localities for *Austrarchaea woodae* highlighted in yellow; **B–C**, topographic and satellite maps showing detail of inset (A). Labelled boundaries in (B) denote upland subregional zones of faunal endemism identified by Winter et al. (1984), Williams et al. (1996) and other authors for the central Wet Tropics (modified from Edward 2011). Small squares in (B–C) denote unidentified juvenile specimens; small circles denote unidentified female specimens; large circles denote described species of *Austrarchaea*. AU = Atherton Uplands; BK = Bellenden Ker/Bartle Frere; HI = Hinchinbrook Island; KU = Kirrama Uplands; LE = Lee Uplands; LU = Lamb Uplands; MT = Malbon-Thompson Uplands.

**Figure 23.**
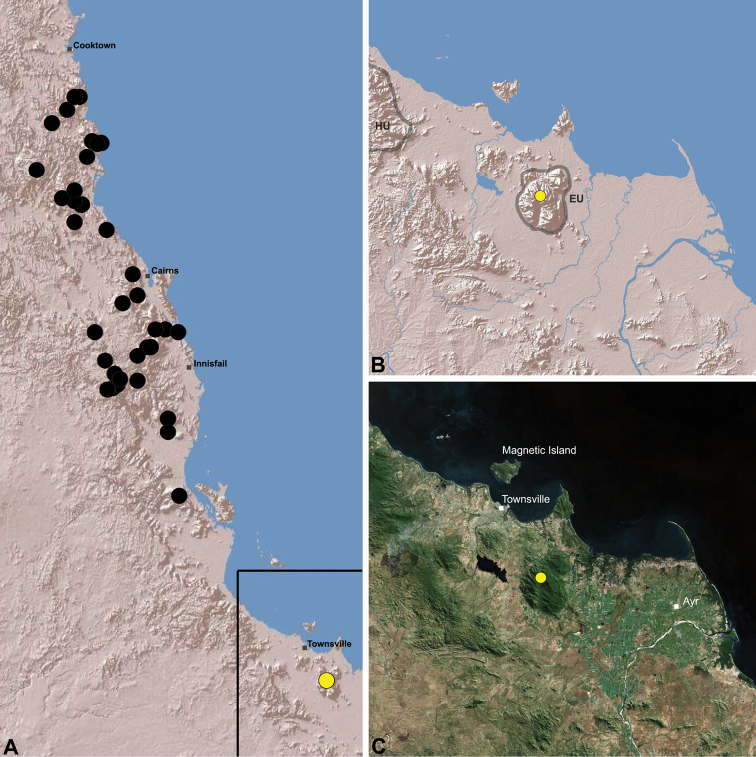
Distribution of *Austrarchaea hoskini* sp. n.: **A**, topographic map showing the known distribution of Archaeidae in the north-eastern Queensland Wet Tropics bioregion, with collection localities for *Austrarchaea hoskini* highlighted in yellow; **B–C**, topographic and satellite maps showing detail of inset (A). Labelled boundaries in (B) denote upland subregional zones of faunal endemism identified by Winter et al. (1984), Williams et al. (1996) and other authors for the southern Wet Tropics (modified from Edward 2011). EU = Elliot Uplands; HU = Halifax Uplands.

**The Wet Tropics World Heritage Area.** The Australian Wet Tropics bioregion, situated in north-eastern Queensland between Cooktown and Townsville (Figs 16–23, 25), is a World Heritage area renowned for its rich rainforest biota and very high levels of local endemism (e.g. see Williams et al. 1996, Crisp et al. 2001, Yeates et al. 2002, Slatyer et al. 2007, and references therein). Much has been written about the biogeography of the region, and numerous seminal contributions over several decades have resulted in the Wet Tropics becoming a model landscape for understanding processes of rainforest biogeography, speciation and diversification, in both plant and animal taxa (e.g. Williams et al. 1996, Schneider et al. 1998, Moritz et al. 2000, Crisp et al. 2001, Yeates et al. 2002). Much of this research has focussed on Pleistocene climatic fluctuations, and the concomitant effects these fluctuations have had on the vicariant biogeography, phylogeography and/or speciation of different taxa, especially vertebrates (e.g. Schneider et al. 1998, Schneider and Moritz 1999, Hoskin et al. 2011, Bell et al. 2012). However, as highlighted by Hoskin et al. (2011), few vertebrate lineages have undergone *in situ* radiation within the Wet Tropics, and most show little phenotypic divergence despite often strong phylogeographic signal; evidence for a deeper and more complex history of speciation. Different taxa also highlight a variety of responses and wildly different patterns of distribution and endemism at different spatial scales (Hoskin et al. 2011), and this is especially true of invertebrates, which often show “extraordinarily high” levels of diversity and endemism compared to vertebrates (Bell et al. 2007: 4995; see also Yeates et al. 2002). Indeed, for flightless or low vagility arthropods, the Wet Tropics have aptly been described as an “epicentre of evolution” (Bouchard 2002: 449), and much remains to be tested in order to understand historical mechanisms of speciation (and subsequent extinction, range contraction or dispersal) in both space and time. Allopatric speciation within Pliocene or Pleistocene refugia has been suggested for at least several endemic insect and vertebrate lineages (e.g. Bell et al. 2007, Hoskin et al. 2011), although deeper, Miocene-age divergences are increasingly being implicated in the major diversification of the Australian Wet Tropics fauna (see Moritz et al. 2000).

Patterns of distribution within the Wet Tropics have historically been assessed in terms of ‘regional endemism’ (i.e. those species confined to the Wet Tropics) versus ‘subregional endemism’ (i.e. those species confined to a single subregion within the Wet Tropics) (Yeates et al. 2002), and an extensive subregional classification has been developed and modified for the entire Wet Tropics over nearly 30 years (e.g. see Winter et al. 1984, Monteith 1995, Williams et al. 1996, Schneider et al. 1998, Moritz et al. 2001, Edward 2011) (Table 1; Figs 16B–23B). This subregional classification, separating adjacent upland and lowland forest blocks, has provided a useful foundation for assessing patterns of distribution, diversity and endemism throughout the Wet Tropics, and has been widely tested or applied in studies of vertebrates (Williams et al. 1996, Schneider et al. 1998, Schneider and Moritz 1999, Dolman and Moritz 2006, Hoskin et al. 2011, Bell et al. 2012) and invertebrates (e.g. Bouchard 2002, Yeates et al. 2002, Bell et al. 2007, Edward 2011, Boyer and Reuter 2012). These studies include comparative analyses of endemicity, biogeographical concordance and conservation significance within and between subregions (e.g. Moritz et al. 2001, Yeates et al. 2002), as well as more traditional estimates of phylogeny, biogeography and phylogeography across a suite of co-occurring taxa. For all such analyses, the historical biogeographic significance of the major upland subregions has been consistently demonstrated, with often strong concordance between palaeoclimatic modelling and phylogeographic structure (Yeates et al. 2002, Hoskin et al. 2011).

**Archaeidae in the Wet Tropics.** Assassin spiders appear to be largely ubiquitous in upland rainforests throughout most of the Wet Tropics, extending from the Finnigan Uplands near Cooktown south to the Elliot Uplands near Townsville (Fig. 25). Archaeidae seem to be less common in lowland tropical rainforests (true of most species of *Austrarchaea* throughout their range), however populations from Mossman Gorge and near Cape Tribulation in the northern Daintree National Park suggest that they may be more widespread in lowland forest systems than current collection records suggest. Indeed, given the relatively high proportion of sites represented only by juvenile specimens or unidentified females (Fig. 25), the tendency for short-range endemism in *Austrarchaea* generally, and the very small number of adult male specimens available for taxonomic research, the Wet Tropics may actually be home to a significantly larger number of archaeid species than documented in this revision. For example, sites like Mount Bartle Frere support two sympatric or at least partially sympatric species (Figs 16, 22), and in the Mossman River region of the southern Daintree National Park, different taxa appear to occupy different lowland (*Austrarchaea tealei*) versus upland (*Austrarchaea thompsoni*) habitats in relatively close proximity (Figs 19–20). Apart from *Austrarchaea daviesae*, which is known from two adjacent subregions (Fig. 16B), all of the other seven species of *Austrarchaea* described from the Wet Tropics are subregional endemics, from the Finnigan Uplands (*Austrarchaea wallacei*), Windsor Uplands (*Austrarchaea karenae*), Carbine Uplands (*Austrarchaea tealei*, *Austrarchaea thompsoni*), Lamb Uplands (*Austrarchaea westi*) and the Bellenden Ker/Bartle Frere Uplands (*Austrarchaea woodae*), respectively (see Table 1).

**Figure 24.**
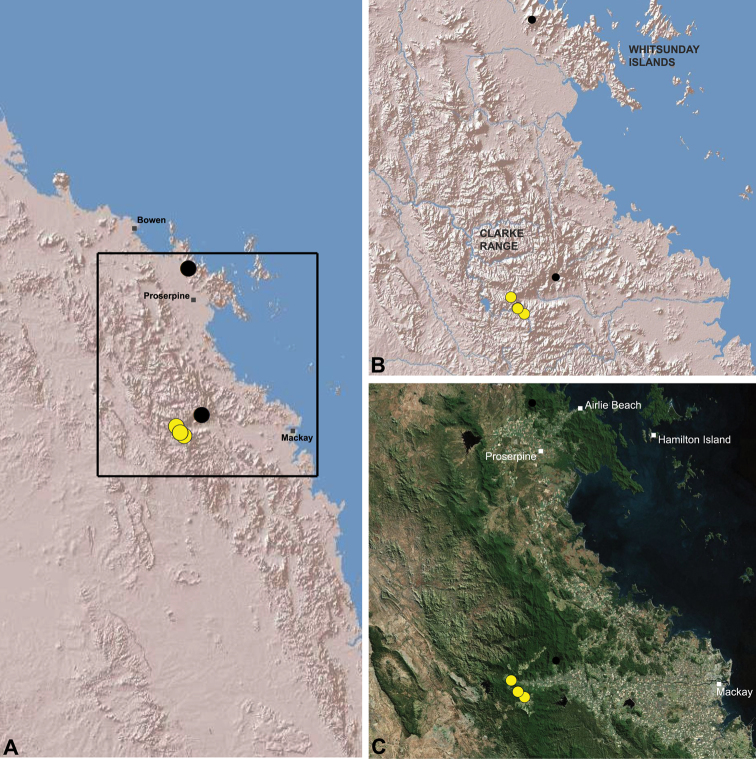
Distribution of *Austrarchaea griswoldi* sp. n.: **A**, topographic map showing the known distribution of Archaeidae in the north-eastern Queensland Mackay and Whitsundays Hinterland, with collection localities for *Austrarchaea griswoldi* highlighted in yellow; **B–C**, topographic and satellite maps showing detail of inset (A). Small circles in (B–C) denote unidentified female specimens; large circles denote described species of *Austrarchaea*.

Estimating the actual number of Archaeidae in north-eastern Queensland is a difficult task, given the surprisingly small number of collection records for the region, the very small number of adult male specimens available, and the related absence in all but two instances of anything other than single-point distributions for most known species (Fig. 25). However, available records do provide some tantalising clues, and hint at the likelihood of a possible hotspot of archaeid diversity in the Wet Tropics. Indeed, with (i) at least four other upland subregional zones with known archaeid records but for which adult male specimens are unavailable, (ii) four additional upland subregions which may harbour archaeid populations but are currently without collections (Table 1), (iii) the likelihood that at least a minority of Wet Tropical subregions may harbour multiple endemic species, either sympatrically or in upland versus lowland habitats, and (iv) the likely presence of at least one additional species in a separate upland zone of the Whitsundays region (Fig. 24), the actual number of taxa in the *Austrarchaea daviesae* species-group is almost certainly > 50% larger than currently recognised. Thus, at a conservative estimate, there may be 15 or more short-range endemic species in tropical Queensland, a number almost equivalent to the total archaeid diversity of mid-eastern Australia. These figures are perhaps not surprising, given the remarkable levels of diversity and endemism seen in other invertebrate groups (see Yeates et al. 2002), but raise the question of how (and when) this diversity was generated. Rix and Harvey (2012b) inferred an Eocene divergence date for taxa in the *Austrarchaea daviesae* species-group (relative to mid-eastern Australian *Austrarchaea*), suggesting that the monophyletic archaeid fauna of north-eastern Queensland has evolved in isolation for 35–50 million years – a result at least consistent with the high levels of interspecific genitalic variation seen across this lineage relative to other Australian clades (Fig. 6). However, geographic sampling for both molecules and morphology is currently inadequate across the Wet Tropics, and a more detailed, thoroughly-sampled molecular study is required to properly assess patterns of speciation in the *Austrarchaea daviesae* species-group, and address the significant gaps in our understanding of divergence dates, distributional boundaries and inter-specific relationships within this lineage. The group’s diversity, strict reliance on rainforest habitats and relative ubiquity in the Wet Tropics certainly makes them an ideal candidate for testing patterns of speciation and biogeography throughout the region, and it is our hope that this revision will provide a solid taxonomic foundation for future research.

## Supplementary Material

XML Treatment for Austrarchaea

XML Treatment for Austrarchaea daviesae

XML Treatment for Austrarchaea wallacei

XML Treatment for Austrarchaea karenae

XML Treatment for Austrarchaea thompsoni

XML Treatment for Austrarchaea tealei

XML Treatment for Austrarchaea westi

XML Treatment for Austrarchaea woodae

XML Treatment for Austrarchaea hoskini

XML Treatment for Austrarchaea

XML Treatment for Austrarchaea griswoldi

XML Treatment for Austrarchaea

## Figures and Tables

**Figure:**
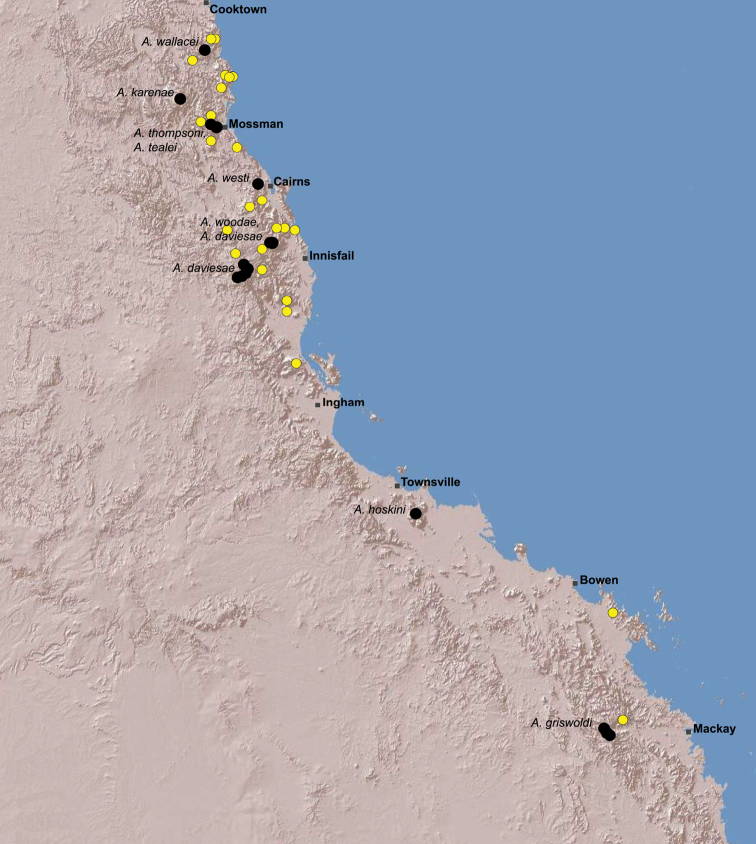
**Figure 25.** Summary distribution of the *Austrarchaea daviesae* species-group in tropical north-eastern Queensland, showing collections records for described species (labelled, with black circles) and unidentified juveniles or females (yellow circles) (see Table 1). Note the high proportion of unidentified specimens, especially within the Wet Tropics bioregion between Cooktown and Ingham.
